# Potential impact of climatic factors on malaria in Rwanda between 2012 and 2021: a time-series analysis

**DOI:** 10.1186/s12936-024-05097-5

**Published:** 2024-09-10

**Authors:** Felix K. Rubuga, Ayman Ahmed, Emmanuel Siddig, Francesco Sera, Giovenale Moirano, Mbituyumuremyi Aimable, Tuyishime Albert, Nshogoza R. Gallican, Eric I. Nebié, Gatera F. Kitema, Penelope Vounatsou, Jürg Utzinger, Guéladio Cissé

**Affiliations:** 1 Swiss Tropical and Public Health Institute, Allschwil, Switzerland; 2 University of Basel, Basel, Switzerland; 3 School of Public Health, College of Medicine and Health Sciences, University of Rwanda, Kigali, Rwanda; 4 Institute of Endemic Diseases, University of Khartoum, Khartoum, Sudan; 5 Department of Medical Microbiology and Infectious Diseases, Erasmus Medical Center, University Medical Center Rotterdam, Rotterdam, The Netherlands; 6 Faculty of Medical Laboratory Sciences, University of Khartoum, Khartoum, Sudan; 7 Department of Statistics, Computer Science and Applications “G. Parenti”, University of Florence, Florence, Italy; 8 University of Turin, Turin, Italy; 9 Rwanda Biomedical Centre, Kigali, Rwanda; 10 Centre de Recherche en Santé de Nouna, Nouna, Burkina Faso; 11 School of Medicine, University of St Andrews, St Andrews, UK; 12 Centre Suisse de Recherches Scientifiques en Côte d’Ivoire, Abidjan, Côte d’Ivoire; 13 Center for Impact, Innovation and Capacity building for Health Information systems and Nutrition (CIIC-HIN), Kigali, Rwanda

**Keywords:** Climatic factors, Location- and season-specific interventions, Malaria, Rwanda, Time-series analysis, Transmission dynamics, Vector surveillance and control

## Abstract

**Background:**

Malaria remains an important public health problem, particularly in sub-Saharan Africa. In Rwanda, where malaria ranks among the leading causes of mortality and morbidity, disease transmission is influenced by climatic factors. However, there is a paucity of studies investigating the link between climate change and malaria dynamics, which hinders the development of effective national malaria response strategies. Addressing this critical gap, this study analyses how climatic factors influence malaria transmission across Rwanda, thereby informing tailored interventions and enhancing disease management frameworks.

**Methods:**

The study analysed the potential impact of temperature and cumulative rainfall on malaria incidence in Rwanda from 2012 to 2021 using meteorological data from the Rwanda Meteorological Agency and malaria case records from the Rwanda Health Management and Information System. The analysis was performed in two stages. First, district-specific generalized linear models with a quasi-Poisson distribution were applied, which were enhanced by distributed lag non-linear models to explore non-linear and lagged effects. Second, random effects multivariate meta-analysis was employed to pool the estimates and to refine them through best linear unbiased predictions.

**Results:**

A 1-month lag with specific temperature and rainfall thresholds influenced malaria incidence across Rwanda. Average temperature of 18.5 °C was associated with higher malaria risk, while temperature above 23.9 °C reduced the risk. Rainfall demonstrated a dual effect on malaria risk: conditions of low (below 73 mm per month) and high (above 223 mm per month) precipitation correlated with lower risk, while moderate rainfall (87 to 223 mm per month) correlated with higher risk. Seasonal patterns showed increased malaria risk during the major rainy season, while the short dry season presented lower risk.

**Conclusion:**

The study underscores the influence of temperature and rainfall on malaria transmission in Rwanda and calls for tailored interventions that are specific to location and season. The findings are crucial for informing policy that enhance preparedness and contribute to malaria elimination efforts. Future research should explore additional ecological and socioeconomic factors and their differential contribution to malaria transmission.

**Supplementary Information:**

The online version contains supplementary material available at 10.1186/s12936-024-05097-5.

## Background

Malaria is a vector-borne parasitic disease. There are five species causing malaria in humans; yet, the majority of cases are attributed to *Plasmodium falciparum* and *Plasmodium vivax* [1]. Competent vectors (several species of *Anopheles* mosquitoes) take up malaria parasites while feeding on the blood of infected humans. Within a few days, the parasites develop and can infect another individual when mosquitoes feed on them. In 2022, the World Health Organization (WHO) estimated that there were 249 million malaria cases and 608,000 deaths worldwide with 94% and 95% of the disease morbidity and mortality concentrated in the African region [2].

Several factors drive the transmission of malaria, including meteo-climatic and environmental factors, unplanned urbanization, inadequate housing, and poor socioeconomic status [3, 4]. A deeper understanding of climate conditions and malaria transmission is important to guide malaria control and elimination efforts [5, 6]. Indeed, variation in climate conditions, such as temperature, rainfall patterns, and humidity, can affect the distribution and dynamics of vector composition, longevity of mosquitoes, the development of malaria parasites within them, and subsequently affecting malaria transmission [7–15].

Furthermore, climate change also plays a role in the geographical shift of *Anopheles* mosquitoes. As temperature and other environmental conditions change, the habitat range of the mosquitoes can expand into previously non-malaria areas [16–22]. This can result in the emergence or re-emergence of malaria in locations where it was absent or previously not a major concern.

In Rwanda, malaria is endemic in 11 of the 30 (37%) districts [23]. The western and northern regions of Rwanda (covering approximately 63% of the surface) are malaria epidemic-prone, while other areas are categorised as endemic and stable malaria transmission. Zones with the major foci are concentrated in the south-eastern and eastern regions [24]. The malaria incidence was 76 cases per 1000 persons-year in 2021–2022, with 1831 severe malaria cases and 71 malaria-related deaths [25]. *Plasmodium falciparum* remains the predominant species (attributable fraction of reported cases: 98%), while the remaining infections are due to *Plasmodium malariae* and *Plasmodium ovale*.

Given the rising temperature and altered patterns of precipitation, climate change might influence the distribution and transmission dynamics of malaria, and hence, pose a threat to malaria control and elimination in Africa, including Rwanda [26, 27]. Despite the critical importance of this issue, there is a paucity of research exploring the relationship between climatic factors and malaria in Rwanda, particularly concerning the use of long-term datasets for time-series analysis [28, 29]. Furthermore, the availability and quality of comprehensive datasets, which integrate both climatic and health-related information, are often limited [22, 30]. This limitation, in turn, restricts the ability to perform detailed time-series analysis, which is essential for understanding how changes in climate variables correlate with fluctuations in malaria incidence.

The main objective of this study was to analyse and quantify the short-term effects of temperature and cumulative rainfall on malaria incidence. A key focus was to explore the potential heterogeneity in these relationships across districts, thereby providing insights for developing localized malaria control and elimination strategies. The analysis aimed to fill existing data gaps and contribute to a more comprehensive understanding of how climatic factors influence malaria transmission within the country.

## Methods

In this study, a two-stage analysis was undertaken to explore the relationship between climatic factors and malaria transmission across Rwanda over a 10-year period from January 2012 to December 2021.

### Study setting

Rwanda is located in eastern Africa, between 1° and 3° S latitude and 29° and 31° E longitude. Rwanda is known as one of the most densely inhabited countries in Africa with an estimated population of 13.2 million and approximately 501 inhabitant per km^2^ [31]. Rwanda is administratively divided into 30 districts in five provinces.

The country’s tropical climate is characterised by distinct geographical and climatic regions, governed by its hilly terrain. It is divided into four climatic areas: eastern plains, central plateau, highlands, and regions around Lake Kivu. The eastern plains exhibit a mean annual temperature of 20–22 °C with a mean annual precipitation of 700–1100 mm. The central plateau has slightly cooler temperatures between 18 °C and 20 °C and slightly more rainfall ranging between 1100 and 1300 mm. The highlands, including the Congo-Nile Ridge and volcanic chains of Birunga, benefit from an annual rainfall of between 1300 and 1600 mm and experience annual mean temperatures between 10 and 18 °C. Regions around Lake Kivu and Bugarama plains get annual rainfall of between 1200 and 1500 mm and annual mean temperatures between 18 and 22 °C. Rwanda experiences four climatic seasons: a long rainy season from March to May, a short rainy season from September to November, a long dry season from June to August, and a short dry season from December to February [26].

Temperature time series suggest that the mean annual temperature has increased by 1.4 °C since 1970, which is considerably higher than the global average [32]. It is expected that the temperature will further increase and reach + 2 °C by the 2030s compared to the 1970s. Analysis of recent rainfall trends suggest an increased occurrence of extreme events (e.g. droughts or floods) in various parts of Rwanda, with eastern regions experiencing serious rainfall deficits over prolonged periods, while there might be rainfall excesses in other years.

### Data collection

This study utilized two primary datasets to explore the relationship between climatic factors and malaria incidence in Rwanda. The meteorological data were obtained from the Rwanda Meteorological Agency (RMA), while health data were obtained from the Rwanda Health Management and Information System (HMIS). In brief, the RMA dataset includes records from January 2012 to December 2021 and corresponds with the epidemiological dataset. The RMA operates 71 meteorological stations strategically positioned across Rwanda, ensuring comprehensive coverage of all climatic zones. These stations routinely gather data on daily parameters such as rainfall, temperature, and relative humidity. For the purpose of this study, monthly metrics were derived, including the average, maximum, and minimum temperature, as well as the total monthly cumulative rainfall, aggregated from daily records.

The HMIS dataset comprises monthly aggregate records of malaria cases spanning from January 1, 2012, to December 31, 2021. The records are both anonymised and aggregated, and collected through the programmatic activities of the routine surveillance. Hence, no personal identifiers were collected, thus ensuring the confidentiality of individual patients’ records. The analysis offers insights into malaria cases at both a national scale and a more granular district level.

### Statistical analysis

In a first step, descriptive analyses on climatic factors and health metrics across different districts were performed. Data were aggregated at the unit of the district and statistical estimates performed for each variable. Specifically, the total cases were computed and the average incidence for malaria calculated. For temperature variables, the mean values for average, maximum, and minimum temperatures were determined. Additionally, the respective ranges, which represent the difference between the highest and lowest recorded temperatures for each category and cumulative rainfall, were calculated. The range helps illustrating the extent of temperature and rainfall variability. The median was ascertained for cumulative rainfall.

Furthermore, data from all 30 districts were analysed, focusing on malaria cases and climatic parameters, including maximum, minimum, and average temperature, as well as cumulative rainfall. To capture the seasonal dynamics, seasonal variations in climatic parameters and malaria cases were plotted, encompassing the four main seasons. A time series for both predictor and outcome variables were plotted at the country level to provide an overall perspective. Subsequently, for each district, time series line plots for all variables were created, offering a district-specific view. A map of average malaria incidence was generated. Scatterplots were developed to illustrate relationships between malaria cases and predictor variables. Additionally, the correlations between temperature variables (minimum, maximum, and average) and rainfall were examined.

For the analysis of the association between climatic parameters and malaria cases across Rwanda, a two-stage design was employed. In the first stage, the estimation of district-specific exposure–response associations between climatic factors and monthly malaria cases were examined. To achieve this, a generalized linear model (GLM) with a quasi-Poisson family was utilized to take into account the possible over-dispersion of malaria cases. For each of the considered exposures (i.e. minimum, maximum, and average temperature, as well as rainfall) distributed lag non-linear models (DLNM) parametrization were employed. These models adeptly capture the potential non-linear and delayed associations between malaria cases and the exposures. For the exposure dimension, a cubic polynomial function was employed. For the lag dimension, a temporal lag of 1 month was considered. To control for potential confounding effects of location-specific trends, a spline function of time with 1 degree of freedom (df) per year was considered. The coefficients of the DLNM parametrization were reduced into the exposure dimension yielding coefficients of the cubic polynomial representing the non-linear association and its variance–covariance matrices for each district [33, 34].

The second stage involved pooling the estimates from the first stage using a random effects multivariate meta-analysis for the association between climatic predictors and malaria cases. Best linear unbiased predictions (BLUPs) were computed to refine first-stage estimates, offering a balance between first-stage district-specific estimates and the pooled mean [35].

In a further step to determine the interplay between climatic factors, each of the temperature-based meta-analytic models were systematically adjusted for cumulative rainfall (see additional files 22 to 27 for more details). Conversely, in examining the relationship between cumulative rainfall and malaria cases, the potential confounding effect of each temperature metric were adjusted for (see additional files 28 to 33 for more details). In a complementary analysis to assess seasonal effects, the same statistical framework was employed but with a modification to the lag structure, setting it to zero. The variable season was stratified into four distinct climatic periods with the long dry season from June to August considered as the reference point. This stratification allowed analysing the direct effects of seasonal changes on malaria transmission. Moreover, a detailed district-specific analysis has been undertaken, exploring the relationships between malaria incidence and various climatic factors, such as temperature (minimum, maximum, and average) and rainfall, across districts. The entire framework of the analysis was implemented using the R packages ‘dlnm’ and ‘mixmeta’ [35–37]. Predictions were generated for pooled, first-stage, and BLUP estimates. The results were illustrated in visual plots showing the relative risk (RR) against minimum temperature, maximum temperature, average temperature, and cumulative rainfall, In contrast to the graphical representation of temperature and rainfall effects, the seasonal effects were quantified in terms of RR and the corresponding confidence intervals (CIs).

### Sensitivity analysis

Lagged effects of climatic variables were explored, examining how changes in temperature or rainfall might influence malaria cases with delays ranging from 1 to 3 months. The selection of this lag is substantiated by the biological mechanism of malaria transmission, including the life cycles of malaria parasites and its vectors, which are potentially influenced by environmental conditions recorded in the previous month(s) [38]. This lag choice ensures the model’s capacity to capture the immediate and delayed effects of climatic variables on mosquito breeding, changes in behaviour, and population size, as well as parasite development, and subsequently malaria transmission to humans. Furthermore, to account for underlying temporal patterns in the data, the spline used for trends were varied, testing between 1 and 4 df (see additional files 2 to 5 for more details).

## Results

Table 1 illustrates the significant variation in climatic factors and malaria incidence across Rwanda from January 2012 to December 2021. In terms of temperature, there was a marked variation across districts. The highest mean maximum temperature was 27.9 °C in Bugesera district, while Nyabihu district experienced the lowest mean maximum at 20.1 °C, indicating a wide range of temperature conditions in different areas. Similarly, average temperatures varied, with some districts like Bugesera, Gatsibo, Nyarugenge, and Kicukiro experiencing mean temperatures around 22 °C, and others like Nyabihu as low as 15.6 °C. Minimum temperatures also showed significant variation, with warmer areas averaging around 16 °C such as Nyarugenge and Kirehe, and cooler areas around 11 °C such as Nyabihu.

In terms of precipitation, as shown in Table 1, the monthly median values for most districts ranged between 60 and 90 mm, with certain districts experiencing lower and higher median rainfall. Against this backdrop of diverse climatic conditions, malaria incidence in Rwanda during the same period showed considerable heterogeneity. Some districts recorded extremely high rates of malaria, while others had notably low incidences, reflecting the complex relationship between environmental factors and health outcomes in different regions of the country.

Detailed results, including specific measures of central tendency and dispersion for temperature and rainfall, as well as malaria incidence rates for each district are available in the additional file 1.

### Overall time series of maximum, minimum, and average temperature, and cumulative rainfall in Rwanda, 2012–2021

**Fig. 1:**
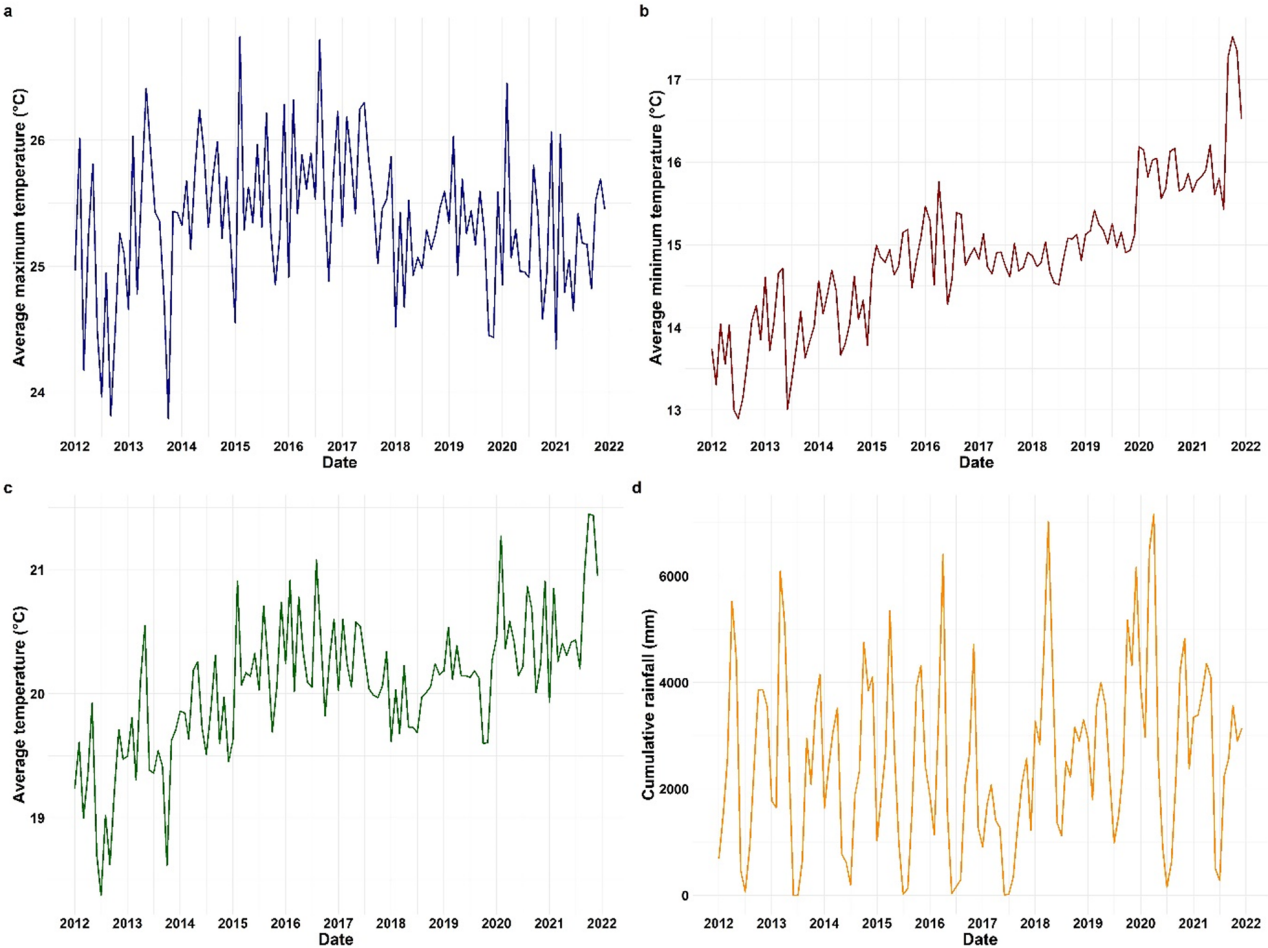
Time series of climatic factors from 2012 to 2021 in Rwanda. **a** Maximum temperature, **b** minimum temperature, **c** average temperature, and **d** rainfall patterns

The analysis showed that malaria-associated meteorological factors, mainly including temperature and rainfall, in Rwanda fluctuated considerably over the 10-year period from 2012 to 2021 (Fig. 1).

### Average incidence of malaria by district in Rwanda, 2012–2021

**Fig. 2:**
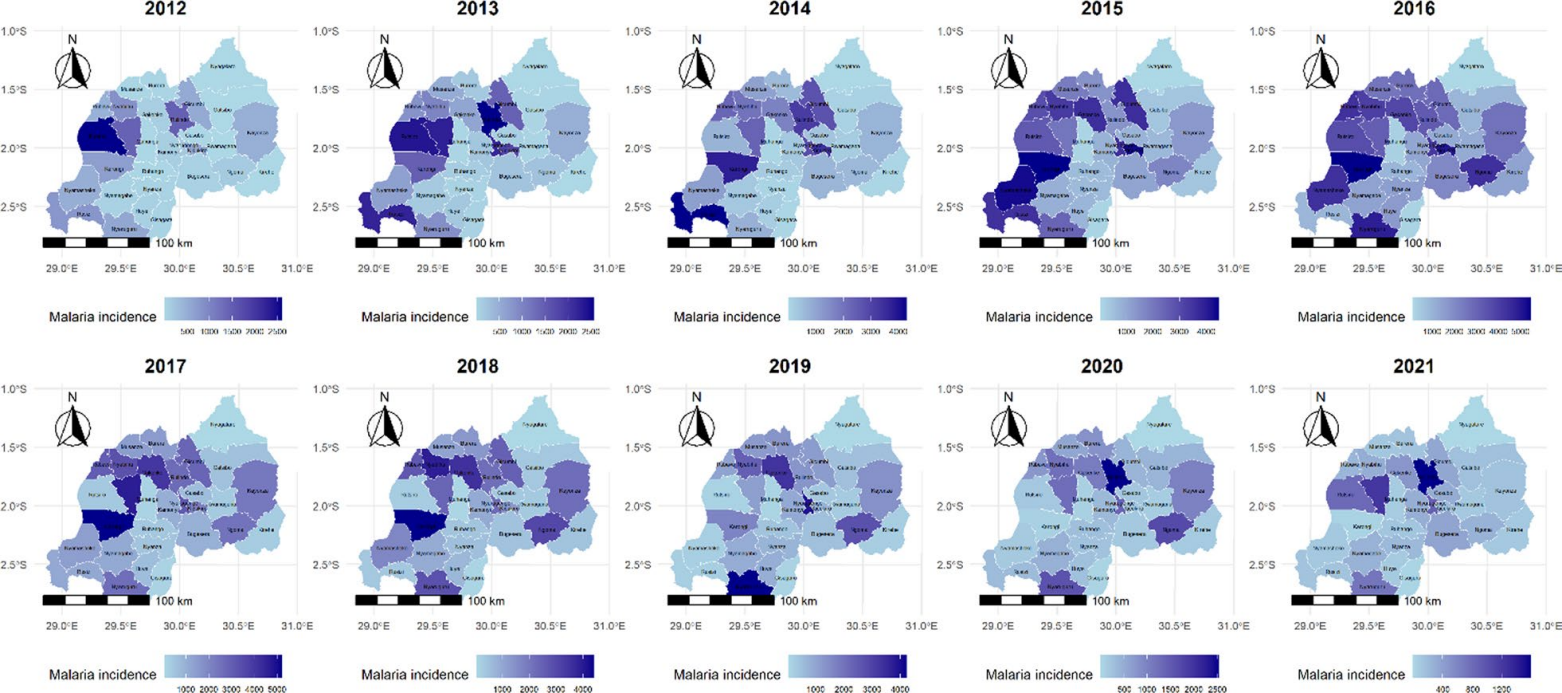
Map of average incidence of malaria by district in Rwanda from 2012 to 2021, expressed as cases per 100,000 per year

Similar to the fluctuation of climate indicators, disparities in malaria incidence rates were recorded across districts in Rwanda from 2012 to 2021 (Fig. 2). Rutsiro registered the highest incidence in 2012, with a peak of 2604 cases per 100,000 inhabitants. Rulindo demonstrated consistently high incidences, escalating from 1461 cases per 100,000 in 2012 to 2569 cases per 100,000 in 2013. Similarly, Ngororero’s malaria incidence rose steadily, reaching a peak of 4663 cases per 100,000 by 2017. Karongi and Nyaruguru also exhibited substantial peaks, with incidences of 5463 and 4840 cases per 100,000, respectively in 2016. Conversely, districts such as Kirehe and Nyagatare had incidences as low as 11.3 and 10.3 cases per 100,000, respectively in 2012, while Rwamagana had an incidence of 13.8 cases per 100,000 the same year.

### Overall time series of malaria cases in Rwanda, 2012–2021

**Fig. 3:**
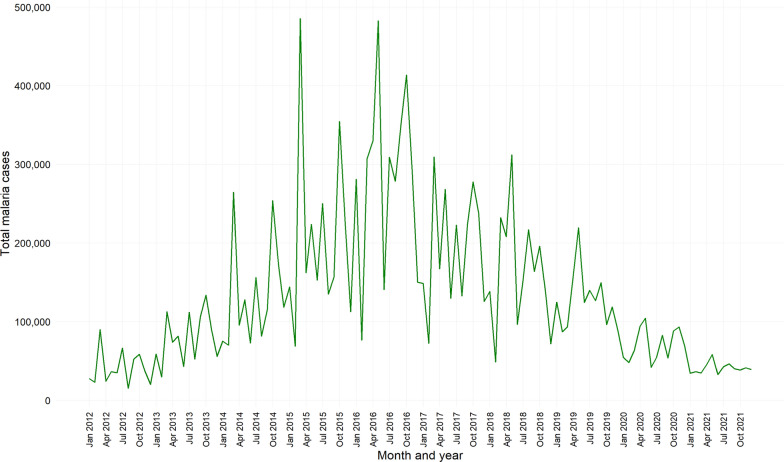
Time series of malaria cases in Rwanda, 2012–2021

In Rwanda, malaria cases have fluctuated over the past decade, but a noticeable increase was reported, especially in early 2015 (Fig. 3). The data suggest a cyclical pattern, with each year marked by peaks and troughs (Fig. 3). Notably, there was a significant spike around mid-2016. Towards the end of the depicted period, a trend of a slight decrease was observed (Fig. 3).

### Seasonal variations in climatic factors and malaria cases

**Fig. 4:**
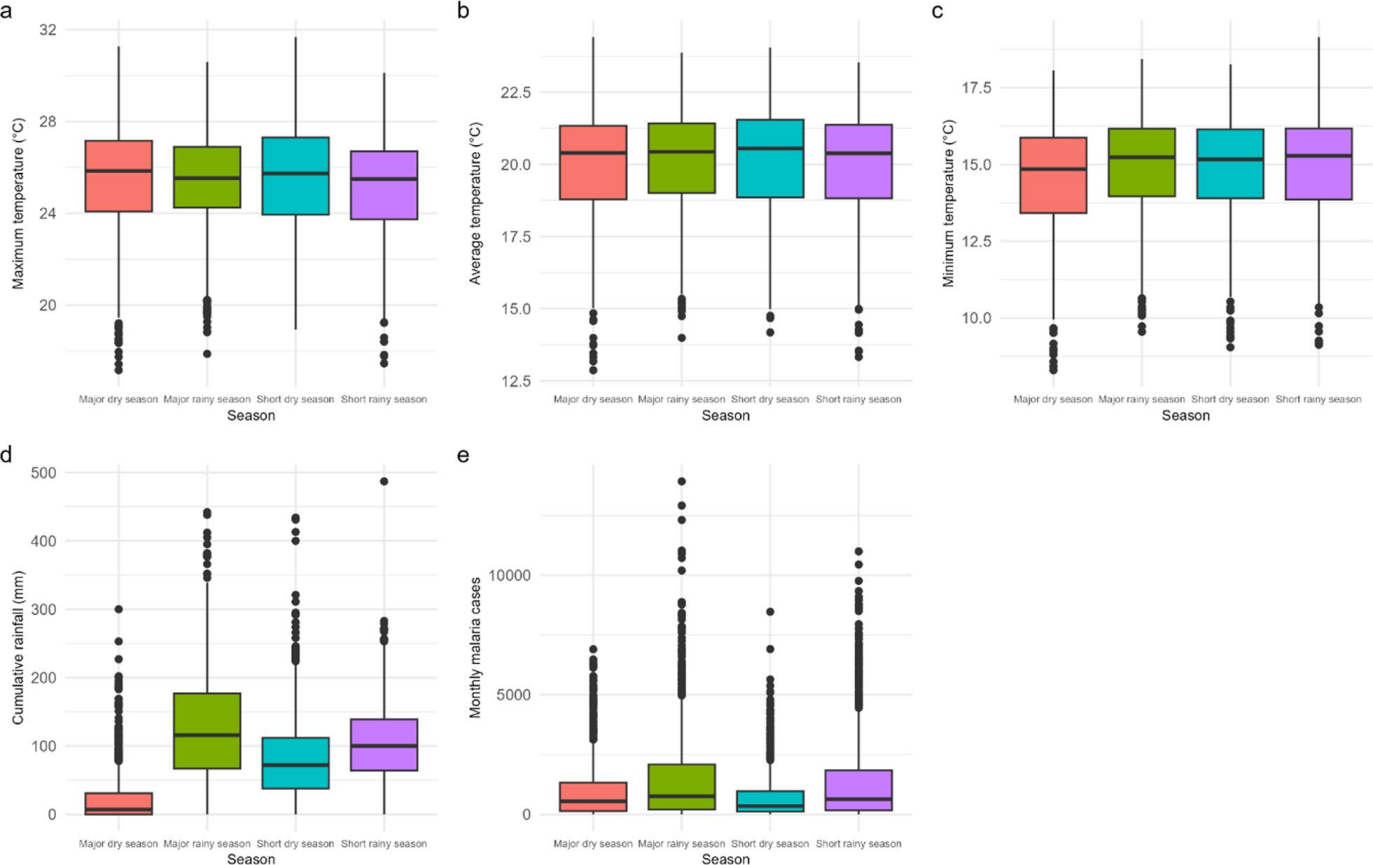
Seasonal variations in weather parameters and malaria cases. **a** Boxplot of maximum temperatures by season, **b** distribution of average temperatures across seasons, **c** trends in minimum temperatures, **d** patterns of cumulative rainfall by season, and **e** monthly malaria cases across different seasons

Data aggregated from the 30 districts of Rwanda were used to describe the seasonal variations in climatic factors and malaria case counts. Temperatures are presented in degrees Celsius (°C) and rainfall in millimetres (mm), as shown in Fig. 4. The maximum temperature (Fig. 4a) exhibits peaks during both the major and short dry seasons. The average temperature (Fig. 4b) and minimum temperatures (Fig. 4c) display relatively consistent patterns across all seasons. The cumulative rainfall (Fig. 4d) reaches its highest levels during the major rainy season, which aligns with the observed spike in malaria cases (Fig. 4e).

### District-specific time series of malaria cases, climatic predictors, and their relationships

The time-series data for malaria cases observed across districts showed a pattern of peaks in 2013, 2014, 2015, and 2016, followed by a decline in 2017. Notably, minimum temperatures have gradually increased over the years with seasonal fluctuations, observed consistently across several districts. Similarly, maximum temperatures also displayed a steady rise across various districts, accompanied by evident seasonal variations. Likewise, average temperatures have consistently risen, tracked across multiple districts with regular seasonal patterns.

Moreover, rainfall data reveal pronounced seasonal peaks and an overall increasing trend across several districts, with higher peaks noted in certain years. This environmental backdrop sets the context for understanding health impacts, as scatterplots relating higher cumulative rainfall to increased malaria cases indicate a consistent positive correlation across various districts. Furthermore, relationships between maximum temperature ranges and malaria cases suggest that certain temperature thresholds commonly associate with increased malaria incidence across multiple districts. Similar observations were made with minimum temperatures, where specific ranges correlate with malaria case counts in various districts, indicating a broader climatic influence on disease prevalence.

Additionally, correlations between average temperature ranges and malaria cases are noted widely, suggesting that moderate climatic conditions may commonly favour malaria transmission. Scatterplots also illustrate correlations between average temperatures and cumulative rainfall, showing that certain temperature ranges are frequently associated with higher rainfall across multiple districts. Similar patterns were observed in relationships between both minimum and maximum temperature ranges with cumulative rainfall, indicating a common trend where temperature extremes coincide with varied rainfall levels across several districts. Detailed district-specific analyses are provided in additional files 6 to 17.

### Association between climatic factors and malaria transmission

In the comprehensive two-stage analysis, the initial phase focused on determining district-specific exposure-lag-response associations using GLM. This model identified the complex non-linear relationships and lagged effects between malaria cases and climatic variables. Subsequently, in the second stage, multivariate meta-analytic techniques were adopted to collate and refine the findings from the first stage.

**Fig. 5:**
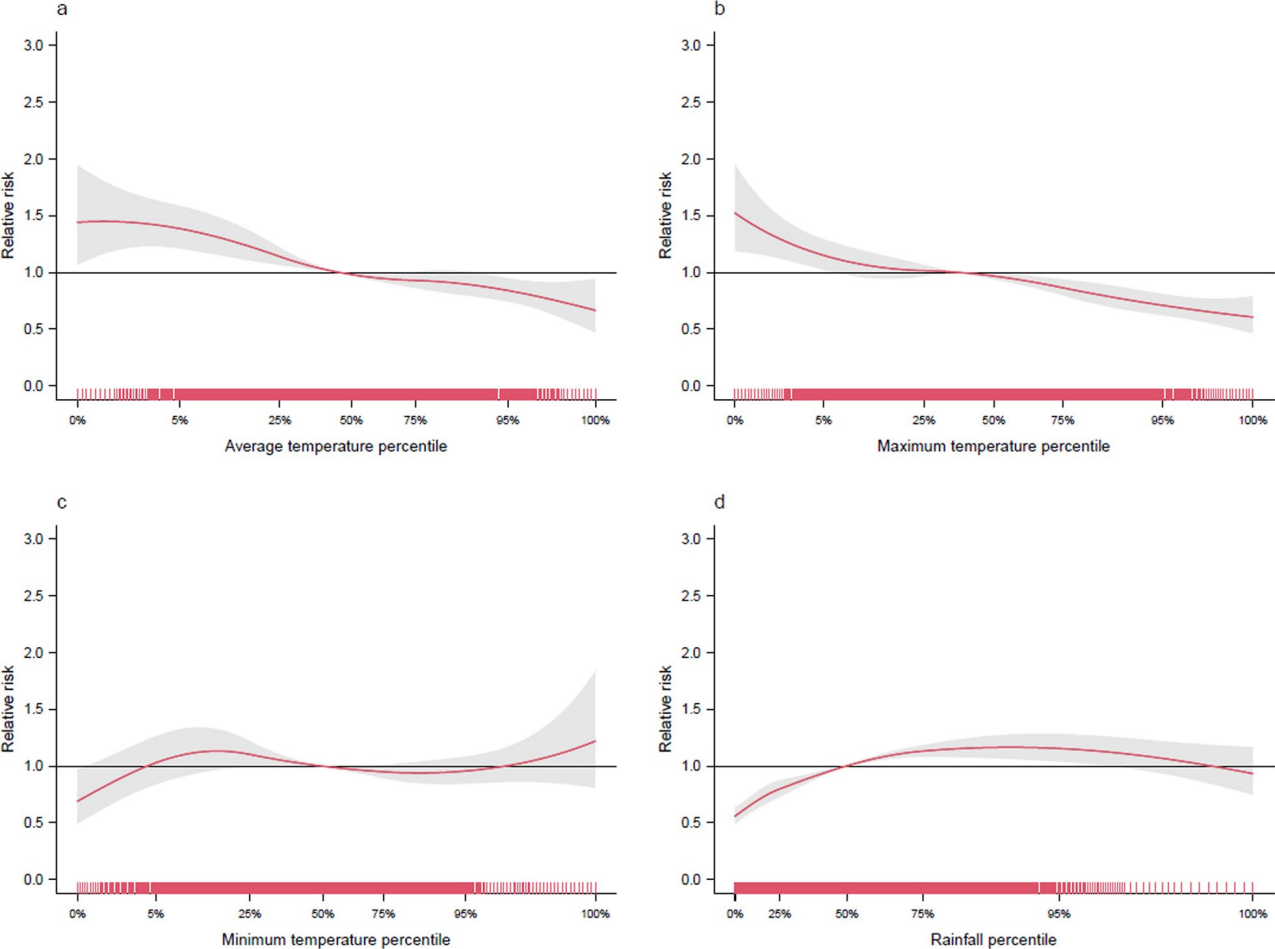
Comprehensive analysis of malaria risk in relation to climatic factors in Rwanda, 2012–2021. Part **a** of the figure illustrates the relationship between average temperature and malaria risk; part **b** shows the impact of maximum temperature on malaria risk; part **c** shifts the focus to the minimum temperature, revealing insights into how lower temperature thresholds affect malaria prevalence; and part **d** examines the relationship between cumulative rainfall and malaria risk

In the combined analysis presented in Fig. 5, the relationships between various temperature measures and cumulative rainfall with malaria risk are systematically explored. Starting with average monthly temperatures (Fig. 5a), a significant increase in malaria risk was observed at lower temperatures, specifically at the 0.2nd percentile (18 °C) with a RR of 1.45 (95% CI: 1.11, 1.89). This trend of increased risk continues up to the 25th percentile (19.5 °C), but beyond the 53.8th percentile (20.1 °C), there was a notable decline in risk.

Turning to maximum monthly temperatures (Fig. 5b), a similar pattern emerged. Increased malaria risk was evident at lower temperatures, particularly below the 5.1st percentile (23.6 °C), with a RR of 1.15 (95% CI: 1.02, 1.29). However, as temperature rise above the 8.4th percentile (23.9 °C), the risk diminishes, indicating a reduced likelihood of malaria with increasing maximum temperatures.

In contrast, the analysis of minimum temperatures (Fig. 5c) revealed a different scenario. The RRs at the 5th (13.1 °C), 75th (15.5 °C), and 95th percentiles (16.4 °C) were not statistically significant, as evidenced by the CIs encompassing the value 1, suggesting no clear effect on malaria risk at these temperature ranges.

Figure 5d examines the impact of cumulative rainfall on malaria risk. A significant increase in malaria cases was observed within a specific rainfall range (88 mm to 223 mm per month), indicating increased risk in moderately wet conditions. However, both drier conditions (below 74 mm per month) and extremely wet conditions (above 223 mm per months) are associated with a reduced risk of malaria.

### District level BLUPS and heterogeneity

The district-level BLUPs were employed to harmonize the variability between individual district estimates and the pooled mean, ensuring a balanced representation. These BLUP-adjusted associations between malaria RR and climatic variables are depicted in additional files 18 to 21.

Concerning heterogeneity across districts, significant variability was observed in the relationship between malaria cases and climatic variables. For average temperature, there was substantial variability (Cochran Q-test p-value < 0.001, *I*^2^ statistic: 63.9%). The associations with maximum temperature also exhibited heterogeneity, with a Cochran Q-test p-value of < 0.001 and an *I*^2^ statistic of 66.9%. Minimum temperature showed notable variability across districts (Cochran Q-test p-value < 0.001, *I*^2^ statistic: 59.9%). Additionally, there was significant variation among districts in the case of cumulative rainfall (Cochran Q-test p-value < 0.001, *I*^2^ statistic: 64.1%).

### Seasonal effect

The multivariate random-effects meta-analysis elucidated a significant seasonal effect on the risk of malaria, considering the major dry season as reference category, the major rainy season was associated with a significant increase in malaria risk (RR: 1.46 (95% CI: 1.39 to 1.54, p < 0.001), indicating a 46% increased risk compared to the major dry season. In contrast, the short dry season was associated with a reduced risk (RR of 0.70; 95% CI: -0.42 to -0.30, p < 0.001), reflecting a 30% decrease in risk relative to the major dry season. The short rainy season was associated with an increased risk (RR of 1.23; 95% CI: 1.14 to 1.32, p < 0.001), or a 23% increase in risk when compared to the major dry season. These statistically significant findings (p < 0.05 for all seasons) are underscored by the substantial heterogeneity indicated by the *I*^2^ statistic of 65.3%.

While the presented results focus on the 1-month lag, it is important to note that the observed RR remained consistent across lags of 1 to 3 months. This consistency validated the decision to emphasise findings associated with a 1-month lag, as it captured the immediate and delayed effects of climatic factors on malaria transmission.

## Discussion

The study provides valuable insights into the relationship between climatic factors and altered risk of malaria transmission. The findings underscore a distinct pattern with regard to temperature and cumulative rainfall reinforcing the role of specific local climatic in determining malaria incidence in Rwanda [39]. As regards temperature, the pooled analysis showed an elevated risk of malaria transmission with decreased average temperature, peaking at around 18 °C and diminished as temperature exceeded 20.1 °C. Nonetheless, a critical observation is the protective effect conferred by maximum temperatures above 24 °C in several districts. Studies in different settings, including South Africa and Botswana, revealed that an increased temperature range between 20 °C and 33 °C could potentially accelerates malaria transmission [8, 40–42]. The observed discrepancy may be attributable to the vector compositions, the evolution adaptability of different population of malaria vectors to local ecological conditions, and bionomics of those vectors [43, 44]. In addition to this direct impact of the climate change on composition, distribution, and behaviour of malaria vectors, there is an indirect role of climate change through shifting the dynamics of human population, which commonly results in malaria outbreaks [45, 46].

As regards rainfall, a dichotomous effect on malaria transmission was observed; moderate rainfall enhances mosquito-breeding habitats, increasing malaria transmission risk, while severe rainfall or arid conditions mitigate this risk. This is because severe weather conditions either disrupts breeding sites through flooding or makes them inhospitable due to drought, leading to reduced vector populations and ultimately lower malaria transmission. In Rwanda, a specific rainfall range has been identified that optimizes conditions for mosquito populations, as indicated in Fig. 5d. This optimal moisture level increases humidity, enhancing the survival and fecundity of adult mosquitoes, which in turn increases their likelihood of multiple feedings and boosts malaria transmission [47]. Conversely, conditions of both excessive rainfall and significant drought result in fewer breeding sites, lowering the risk of malaria. Excessive rainfall disrupts larval habitats, while drought leads to their desiccation [48–50]. These findings mirror patterns observed in studies from other African countries, including Botswana, Ethiopia, Ghana, Kenya, Mozambique, and South Africa, highlighting a consistent trend of the impact of rainfall on malaria dynamics [8, 40–42, 51–57].

Furthermore, another layer of complexity arises from district heterogeneity in malaria RR in correlation with climate variables. This variation can be attributed to a wide range of factors, including disparities in health care provision between rural and urban areas, development of resistance among malaria vectors, and climatic change. Additionally, socioeconomic and ecological factors, such as changing land use patterns, particularly the increase in rice farming further influence these dynamics. These elements not only affect the effectiveness of the implemented interventions but also the uptake of preventive public health measures by the local communities [6, 58–63].

Additionally, these results underscore pronounced seasonal fluctuations in malaria risk, tightly correlated with climatic changes, particularly the distinct wet and dry seasons in Rwanda. This seasonal pattern suggests a dynamic interaction between climatic conditions and malaria transmission cycles, necessitating adaptive management strategies that account for seasonal variability [57]. The findings further reveal that these environmental shifts lead to variations in mosquito populations, thereby influencing malaria transmission. Specifically, the unique weather pattern of Rwanda, characterised by alternating periods of rain and dryness, enhances the effect of seasonal variation on malaria transmission. This cyclical climate allows mosquito populations to maintain persistent transmission capabilities throughout the year [64, 65].

## Strengths and limitations

In this study, several attributes were present that added credibility and depth to the findings. Central to the research was the utilization of two robust datasets. The meteorological data from RMA provided a rich, decade-long insight into Rwanda’s climatic patterns. Complementing this was the extensive overview of malaria cases from the HMIS, covering the same period. This combination allowed for a detailed analysis, yielding insights that were nationally relevant and specific to all 30 districts of Rwanda. The granularity of this approach was immensely valuable, particularly for targeted public health interventions. Ensuring the confidentiality of individual patients was a paramount concern, achieved by focusing exclusively on anonymized and aggregated health records obtained through the programmatic routine surveillance system. Methodologically, the two-stage analysis, supported by multivariate meta-analysis techniques, facilitated a detailed exploration of the relationship between meteorological dynamics and malaria case counts. The temporal span of the datasets, encompassing a decade, ensured the identification of both immediate fluctuations and more prolonged trends in the interaction between meteorological patterns and malaria.

The findings of this study illuminate the interplay between climatic factors and malaria incidence. The observed associations between temperature, rainfall variations, and malaria risk underscore the role of climatic factors in influencing the dynamics of malaria transmission. Such insights are invaluable for both national malaria control programme managers and policymakers. For the national malaria control programme, understanding these associations can help predict potential outbreaks and prepare healthcare systems accordingly, ensuring timely interventions and efficient allocation of resources. On the other hand, for policymakers, the results emphasise the importance of integrating climatic data into public health strategies. By doing so, policymakers can formulate and implement more effective and timely preventive measures, especially in districts where climatic fluctuations are pronounced. Furthermore, it highlights the need for district-specific health campaigns, infrastructure development, and education programmes to safeguard communities against potential malaria outbreaks associated with changing climate patterns.

Several limitations are offered for discussion. A major shortcoming was the use of monthly data, which lacked the granularity of daily data, potentially limiting the depth of analysis. Moreover, the study did not account for changes in land use, water bodies, vegetation cover, and other relevant environmental factors known to influence malaria transmission dynamics. Although the heterogeneity across districts was highlighted, the specific factors underlying these differences remained unexplored. Another limitation was the absence of a detailed demographic analysis. The extensive nature of the dataset notwithstanding, the lack of detailed demographic information restricted the ability to conduct a thorough analysis based on factors such as age, sex, or other demographic features. Such an analysis could have provided deeper insights, particularly in identifying population groups at the highest risk of malaria. This gap underscores the importance of incorporating comprehensive demographic data in future research. Addressing these limitations in future research is essential for a more nuanced understanding of the complex interplay between climatic factors and the incidence of malaria, crucial for developing tailored public health interventions.

## Conclusion

The study revealed an association between temperature and cumulative rainfall with malaria transmission in Rwanda. It uncovered how temperature and rainfall influence malaria spread, with specific thresholds showing a notable impact on disease dynamics. The study also found that higher maximum temperature might have a protective effect against malaria. The study noted a complex interaction between rainfall patterns and malaria incidence, with drier conditions reducing risk and certain rainfall ranges escalating it. Importantly, the research also revealed that during the major and short rainy seasons, there was a marked increase in malaria risk compared to the dry season. Significant variability in the malaria-climate relationship was observed across Rwanda’s districts. This heterogeneity indicated that climatic variables impact on malaria transmission and they varied from one district to another, emphasising the necessity for localized interventions tailored to the districts unique climate and epidemiology. This research contributes to global epidemiological knowledge, offering specific insights for Rwanda. It underscores that a one-size-fits-all approach for malaria control and elimination may not be effective in Rwanda. Instead, integrating these district-specific findings into national policies and action strategies is vital for Rwanda’s targeted malaria management efforts.

## Supplementary Information

Supplementary Material 1.

Supplementary Material 2.

Supplementary Material 3.

Supplementary Material 4.

Supplementary Material 5.

Supplementary Material 6.

Supplementary Material 7.

Supplementary Material 8.

Supplementary Material 9.

Supplementary Material 10.

Supplementary Material 11.

Supplementary Material 12.

Supplementary Material 13.

Supplementary Material 14.

Supplementary Material 15.

Supplementary Material 16.

Supplementary Material 17.

Supplementary Material 18.

Supplementary Material 19.

Supplementary Material 20.

Supplementary Material 21.

Supplementary Material 22.

Supplementary Material 23.

Supplementary Material 24.

Supplementary Material 25.

Supplementary Material 26.

Supplementary Material 27.

Supplementary Material 28.

Supplementary Material 29.

Supplementary Material 30.

Supplementary Material 31.

Supplementary Material 32.

Supplementary Material 33.

## Data Availability

No datasets were generated or analysed during the current study.
