# Supplementary material for: Potential impact of climatic factors on malaria in Rwanda between 2012 and 2021: a time-series analysis
Source: Malar J. 2024 Sep 10;23:274. doi: 10.1186/s12936-024-05097-5 (PMC11389490; doi:10.1186/s12936-024-05097-5)
Supplement: Supplementary file 1 — Supplementary Material 1. [file 12936_2024_5097_MOESM1_ESM.pdf]

Table 1: District level summary statistics of temperature, rainfall and malaria (measures of central tendency and dispersion) in Rwanda, 2012-2021

District	Monthly average temperature (°C)	Range average temperature (°C)	Monthly maximum temperature (°C)	Range maximum temperature (°C)	Monthly minimum temperature (°C)	Range minimum temperature (°C)	Monthly median rain (mm)	Range rain (mm)	Total malaria	Average monthly incidence per 100,000
Bugesera	21.9	4.7	27.9	5.3	15.9	5.6	39.2	279	352,701	661
Burera	17.7	4.2	21.9	5.0	13.6	5.7	89.0	310	437,891	1,006
Gakenke	20.2	4.2	25.8	5.4	14.7	5.2	66.8	412	940,697	2,221
Gasabo	21.5	3.7	26.9	5.0	16.0	5.1	98.8	382	362,883	439
Gatsibo	21.9	4.1	27.7	6.0	16.2	6.2	55.5	487	210,823	354
Gicumbi	17.8	3.7	22.3	4.7	13.3	5.1	83.2	311	923,842	1,837
Gisagara	20.6	3.4	26.2	4.8	15.0	4.5	66.8	417	43,712	102
Huye	20.4	3.9	25.5	4.9	15.2	4.8	71.4	377	228,293	536
Kamonyi	21.0	3.5	26.4	6.0	15.5	5.9	66.6	352	297,214	626
Karongi	20.8	4.0	26.1	4.0	15.4	6.8	91.0	292	1,144,846	2,725
Kayonza	21.3	4.1	26.9	6.0	15.6	5.1	71.6	267	610,999	1,272
Kicukiro	21.9	4.1	27.5	4.7	16.3	4.9	64.8	369	898,310	1,974
Kirehe	21.4	4.1	26.6	6.3	16.3	4.2	36.4	226	150,243	313
Muhanga	19.9	4.7	25.0	5.1	14.9	7.0	65.6	442	42,993	105
Musanze	18.1	4.5	23.7	4.8	12.6	6.1	89.0	267	526,561	1,050
Ngoma	21.4	3.4	26.7	4.6	16.1	5.8	60.0	288	771,882	1,725
Ngororero	21.0	3.7	25.8	6.3	16.2	4.6	75.2	339	920,225	2,192
Nyabihu	15.6	4.8	20.1	5.5	11.0	6.2	85.1	366	821,478	2,233
Nyagatare	20.9	4.5	26.8	5.7	14.9	6.9	47.2	234	24,182	37
Nyamagabe	18.3	3.3	23.0	4.4	13.6	5.5	89.5	387	277,134	647
Nyamasheke	20.8	2.9	26.3	4.8	15.2	4.3	97.5	288	700,924	1,450
Nyanza	19.8	3.4	25.7	6.0	13.8	4.2	78.0	346	188,887	456
Nyarugenge	21.9	4.0	27.5	4.9	16.4	4.3	71.3	392	957,710	2,438
Nyaruguru	19.4	4.1	24.8	5.1	13.9	5.9	86.6	400	791,597	2,145
Rubavu	20.2	2.9	25.0	3.5	15.4	5.0	86.4	230	1,037,919	1,855

Ruhango	19.8	4.0	24.8	5.0	14.8	6.4	66.4	420	149,479	366
Rulindo	19.2	4.9	24.1	6.2	14.4	6.0	56.1	424	856,796	2,218
Rusizi	20.8	3.0	26.1	3.9	15.5	4.8	89.5	239	711,265	1,391
Rutsiro	18.6	7.0	23.5	7.0	13.7	8.3	106.3	291	560,630	1,378
Rwamagana	21.1	4.1	26.5	5.8	15.8	5.9	49.2	337	157,871	337
