# Supplementary material for: Potential impact of climatic factors on malaria in Rwanda between 2012 and 2021: a time-series analysis
Source: Malar J. 2024 Sep 10;23:274. doi: 10.1186/s12936-024-05097-5 (PMC11389490; doi:10.1186/s12936-024-05097-5)
Supplement: Supplementary file 2 — Supplementary Material 2. [file 12936_2024_5097_MOESM2_ESM.pdf]

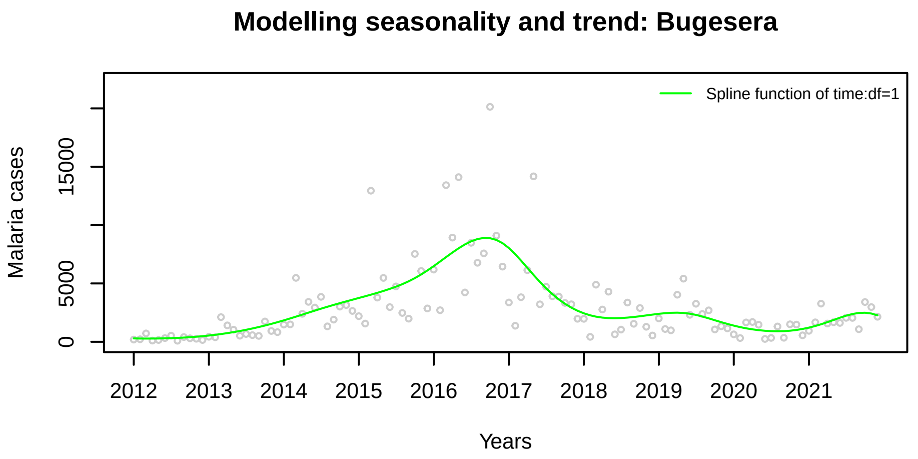
Modelling seasonality and trend: Bugesera
Spline function of time:df=1
Malaria cases
0
5000
10000
15000
2012
2013
2014
2015
2016
2017
2018
2019
2020
2021
Years

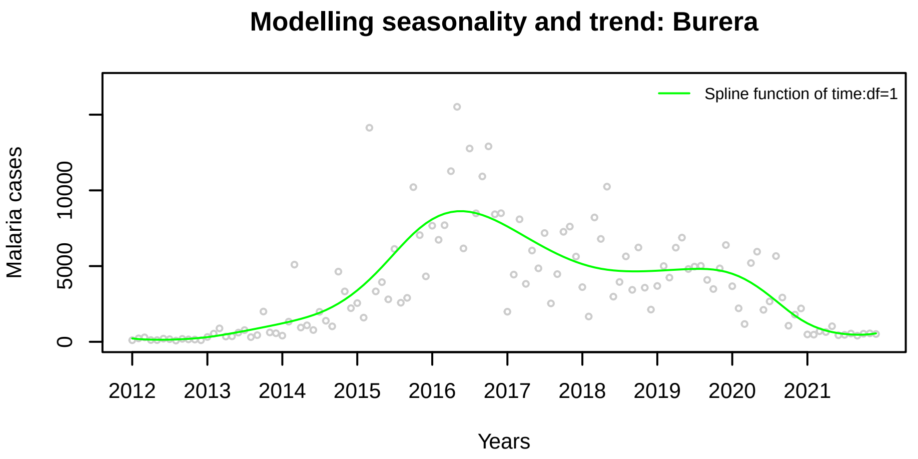
Modelling seasonality and trend: Burera
Spline function of time:df=1
Malaria cases
0
5000
10000
2012
2013
2014
2015
2016
2017
2018
2019
2020
2021
Years

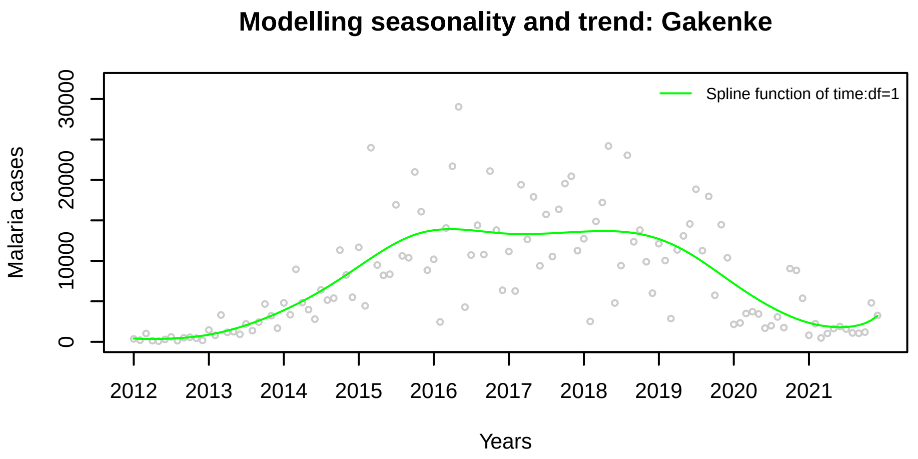
Modelling seasonality and trend: Gakenke
Spline function of time:df=1
Malaria cases
0
10000
20000
30000
2012
2013
2014
2015
2016
2017
2018
2019
2020
2021
Years

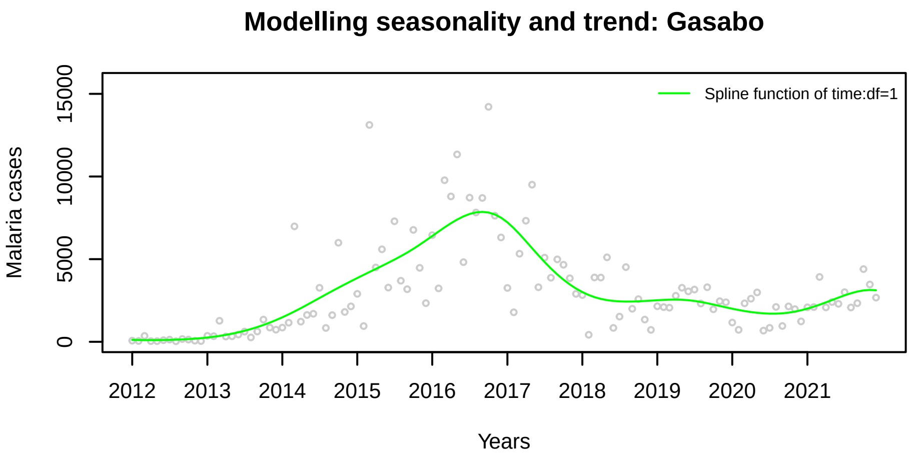
Modelling seasonality and trend: Gasabo
Spline function of time:df=1
Malaria cases
0
5000
10000
15000
2012
2013
2014
2015
2016
2017
2018
2019
2020
2021
Years

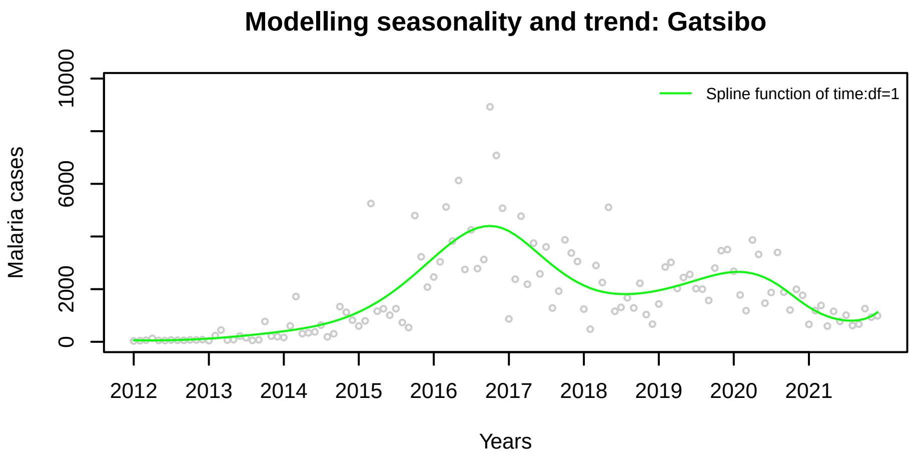
Modelling seasonality and trend: Gatsibo
Spline function of time:df=1
Malaria cases
0
2000
6000
10000
2012
2013
2014
2015
2016
2017
2018
2019
2020
2021
Years

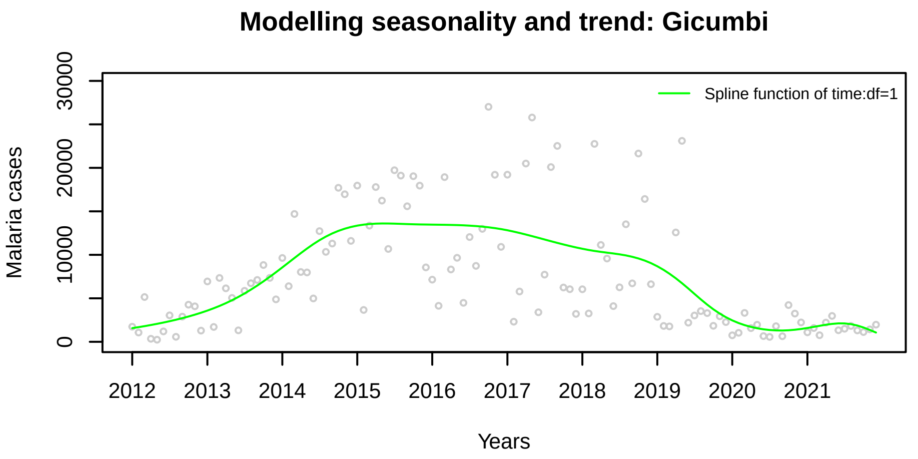
Modelling seasonality and trend: Gicumbi
Spline function of time:df=1
Malaria cases
0
10000
20000
30000
2012
2013
2014
2015
2016
2017
2018
2019
2020
2021
Years

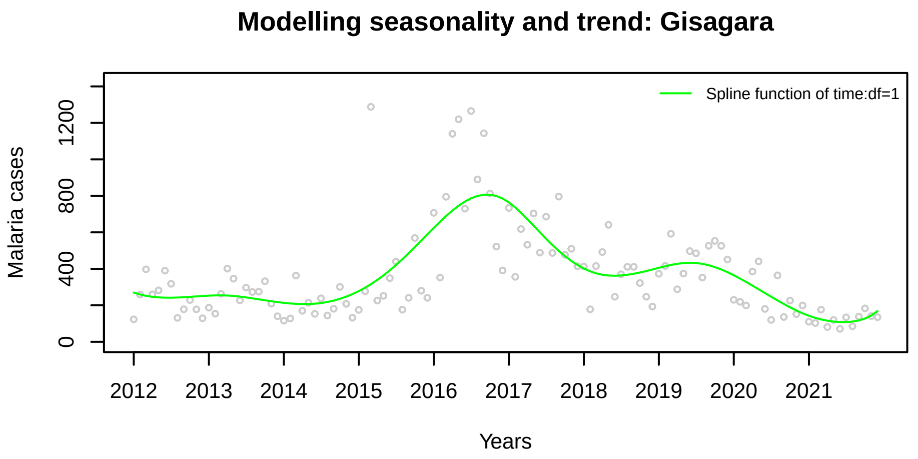
Modelling seasonality and trend: Gisagara
Spline function of time:df=1
Malaria cases
0
400
800
1200
2012 2013 2014 2015 2016 2017 2018 2019 2020 2021
Years

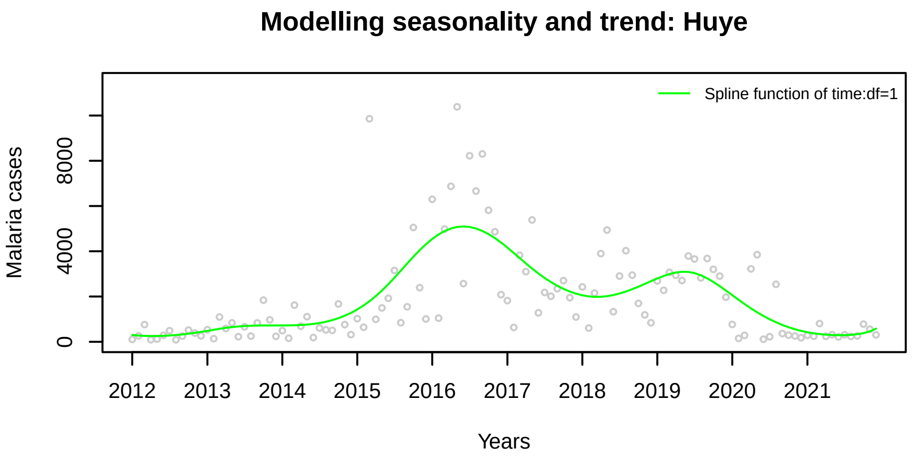
Modelling seasonality and trend: Huye
Spline function of time:df=1
Malaria cases
0
4000
8000
2012 2013 2014 2015 2016 2017 2018 2019 2020 2021
Years

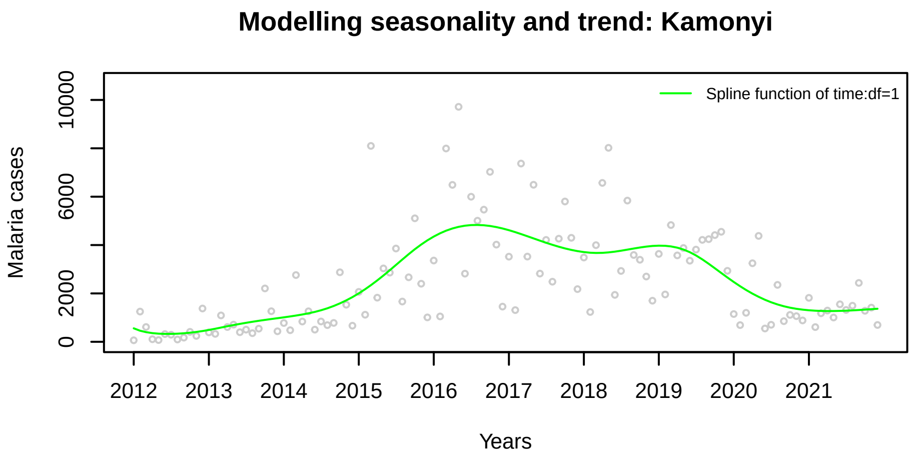
Modelling seasonality and trend: Kamonyi
Spline function of time:df=1
Malaria cases
0
2000
6000
10000
2012 2013 2014 2015 2016 2017 2018 2019 2020 2021
Years

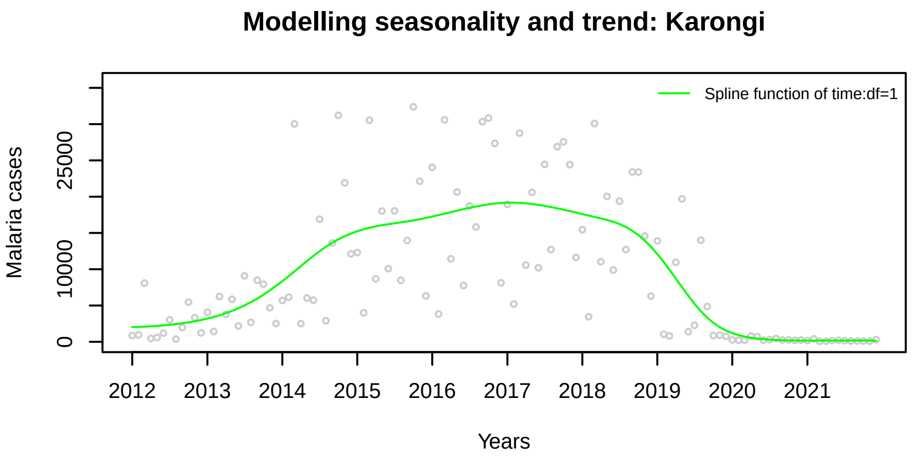
Modelling seasonality and trend: Karongi
Spline function of time:df=1
Malaria cases
0
10000
25000
2012 2013 2014 2015 2016 2017 2018 2019 2020 2021
Years

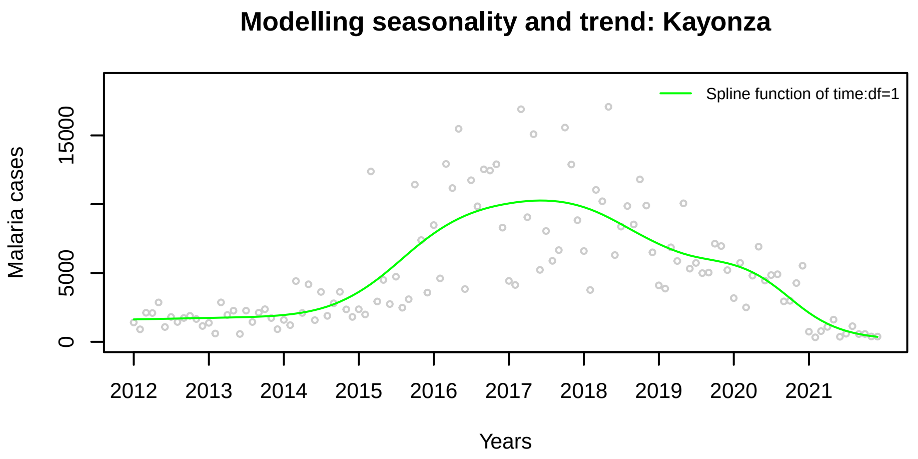
Modelling seasonality and trend: Kayonza
Spline function of time:df=1
Malaria cases
0
5000
15000
2012 2013 2014 2015 2016 2017 2018 2019 2020 2021
Years

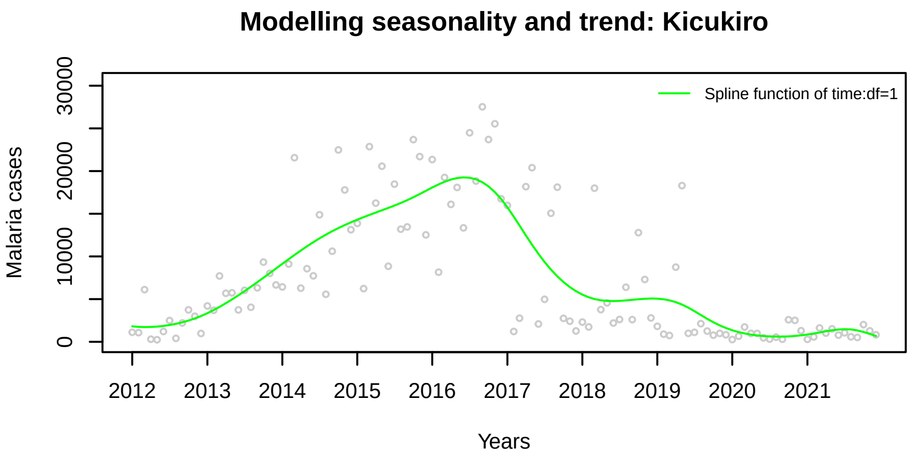
Modelling seasonality and trend: Kicukiro
Spline function of time:df=1
Malaria cases
0
10000
20000
30000
2012 2013 2014 2015 2016 2017 2018 2019 2020 2021
Years

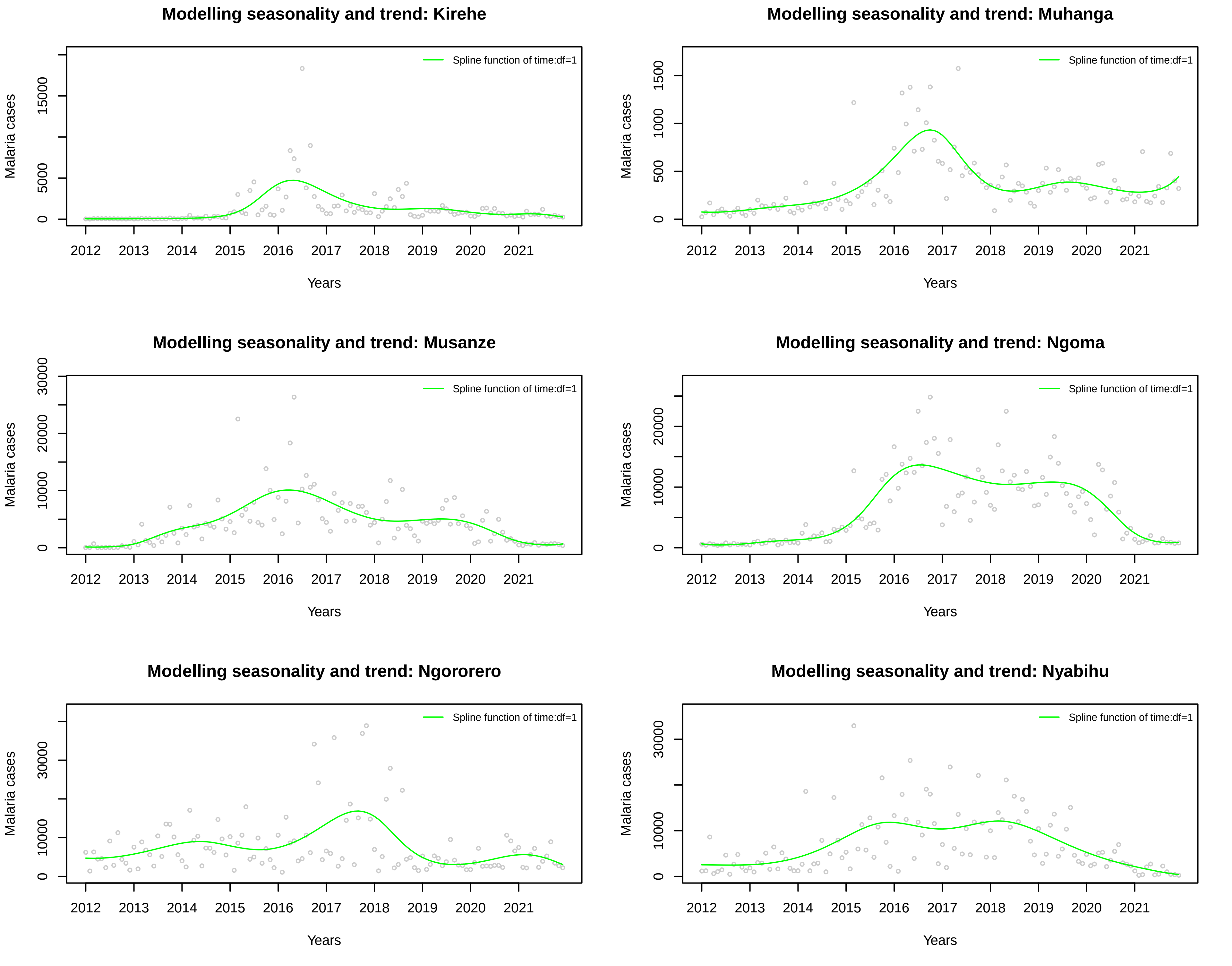
Modelling seasonality and trend: Kirehe
Spline function of time:df=1
Malaria cases
0
5000
15000
2012
2013
2014
2015
2016
2017
2018
2019
2020
2021
Years
Modelling seasonality and trend: Muhanga
Spline function of time:df=1
Malaria cases
0
500
1000
1500
2012
2013
2014
2015
2016
2017
2018
2019
2020
2021
Years
Modelling seasonality and trend: Musanze
Spline function of time:df=1
Malaria cases
0
10000
20000
30000
2012
2013
2014
2015
2016
2017
2018
2019
2020
2021
Years
Modelling seasonality and trend: Ngoma
Spline function of time:df=1
Malaria cases
0
10000
20000
2012
2013
2014
2015
2016
2017
2018
2019
2020
2021
Years
Modelling seasonality and trend: Ngororero
Spline function of time:df=1
Malaria cases
0
10000
30000
2012
2013
2014
2015
2016
2017
2018
2019
2020
2021
Years
Modelling seasonality and trend: Nyabihu
Spline function of time:df=1
Malaria cases
0
10000
30000
2012
2013
2014
2015
2016
2017
2018
2019
2020
2021
Years

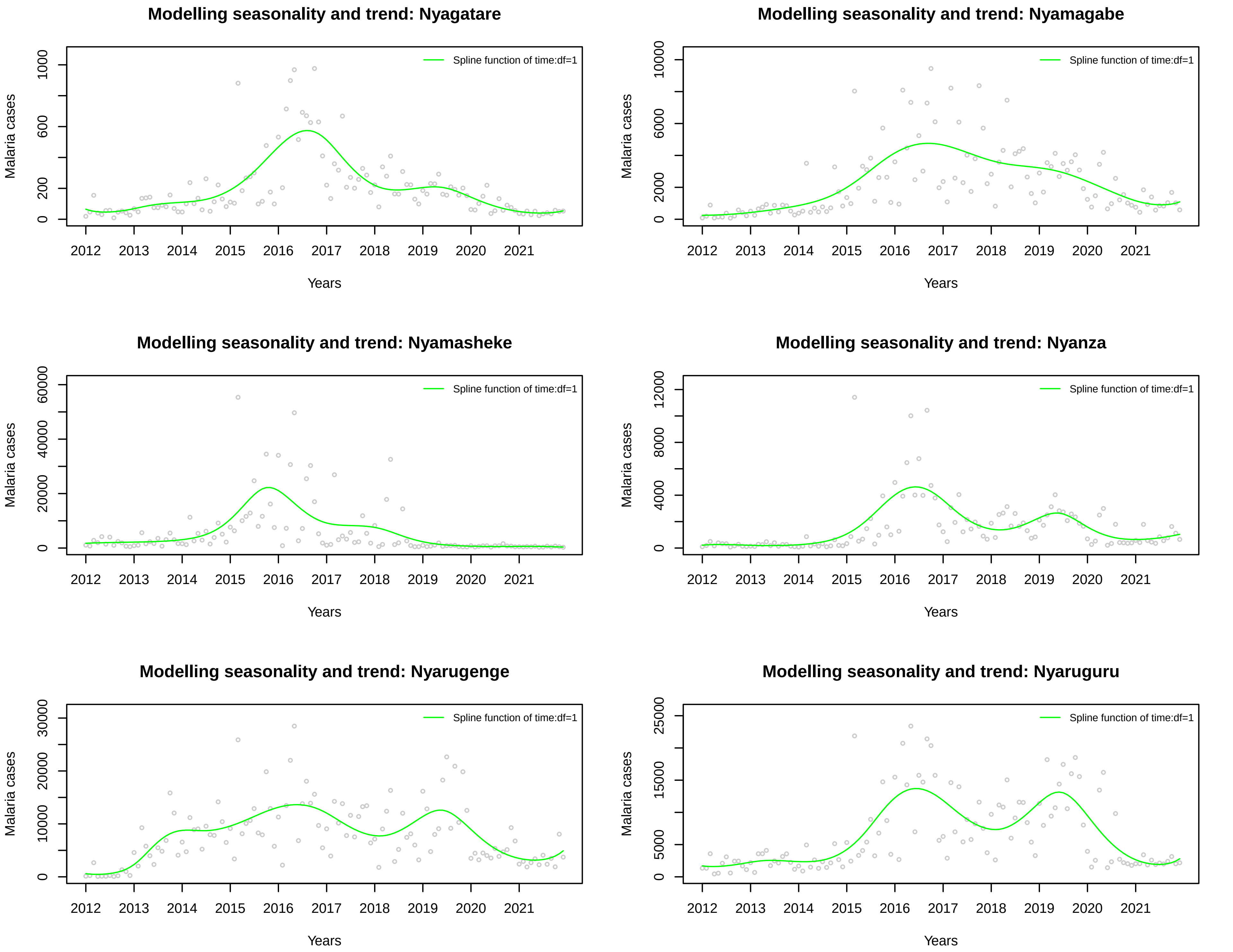
Modelling seasonality and trend: Nyagatare
Spline function of time:df=1
Malaria cases
Years
Modelling seasonality and trend: Nyamagabe
Spline function of time:df=1
Malaria cases
Years
Modelling seasonality and trend: Nyamasheke
Spline function of time:df=1
Malaria cases
Years
Modelling seasonality and trend: Nyanza
Spline function of time:df=1
Malaria cases
Years
Modelling seasonality and trend: Nyarugenge
Spline function of time:df=1
Malaria cases
Years
Modelling seasonality and trend: Nyaruguru
Spline function of time:df=1
Malaria cases
Years
2012 2013 2014 2015 2016 2017 2018 2019 2020 2021

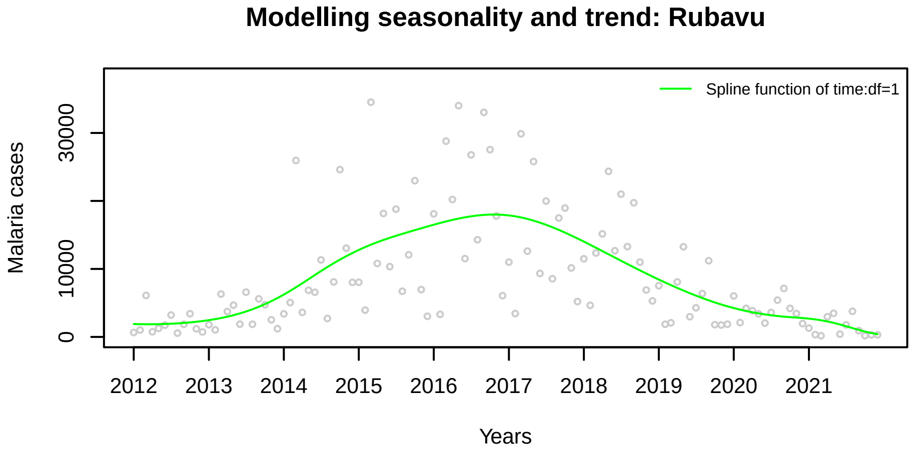
Modelling seasonality and trend: Rubavu
Spline function of time:df=1
Malaria cases
0
10000
30000
2012
2013
2014
2015
2016
2017
2018
2019
2020
2021
Years

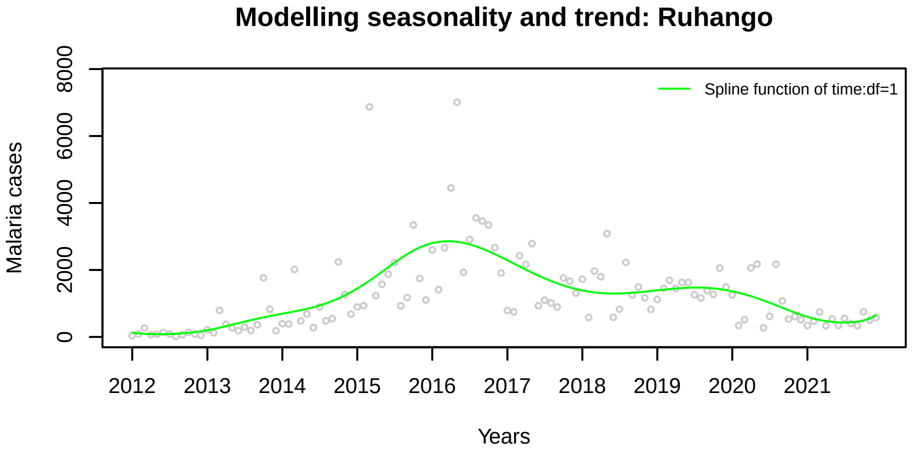
Modelling seasonality and trend: Ruhango
Spline function of time:df=1
Malaria cases
0
2000
4000
6000
8000
2012
2013
2014
2015
2016
2017
2018
2019
2020
2021
Years

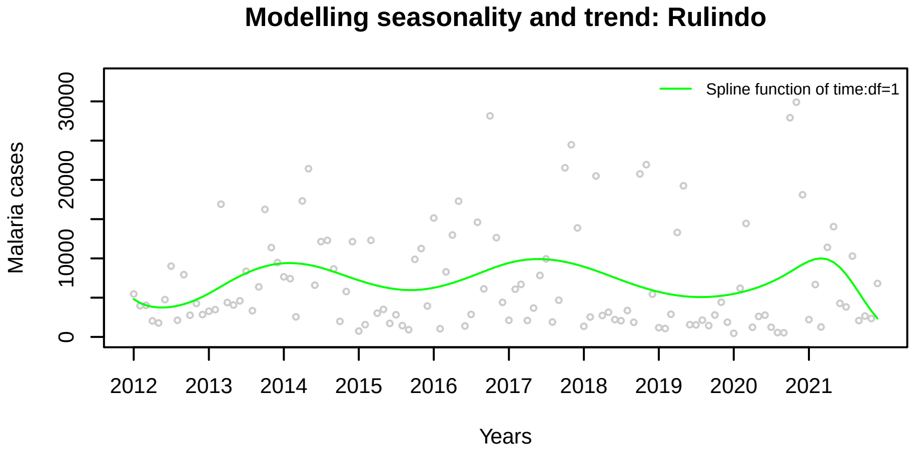
Modelling seasonality and trend: Rulindo
Spline function of time:df=1
Malaria cases
0
10000
20000
30000
2012
2013
2014
2015
2016
2017
2018
2019
2020
2021
Years

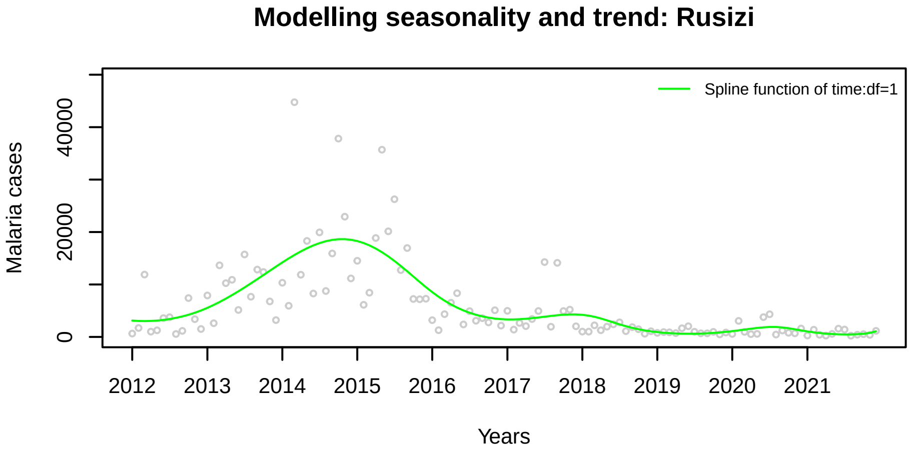
Modelling seasonality and trend: Rusizi
Spline function of time:df=1
Malaria cases
0
20000
40000
2012
2013
2014
2015
2016
2017
2018
2019
2020
2021
Years

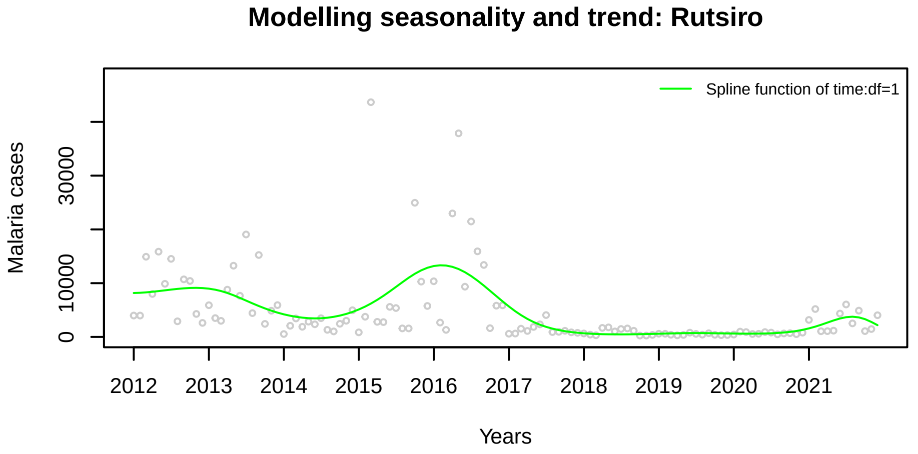
Modelling seasonality and trend: Rutsiro
Spline function of time:df=1
Malaria cases
0
10000
30000
2012
2013
2014
2015
2016
2017
2018
2019
2020
2021
Years

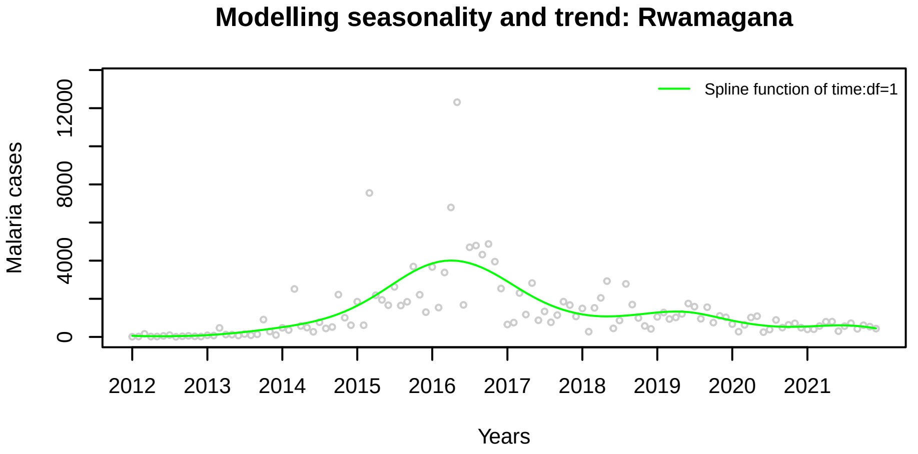
Modelling seasonality and trend: Rwamagana
Spline function of time:df=1
Malaria cases
0
4000
8000
12000
2012
2013
2014
2015
2016
2017
2018
2019
2020
2021
Years
