# Supplementary figures and images for: Potential impact of climatic factors on malaria in Rwanda between 2012 and 2021: a time-series analysis
Source: Malar J. 2024 Sep 10;23:274. doi: 10.1186/s12936-024-05097-5 (PMC11389490; doi:10.1186/s12936-024-05097-5)

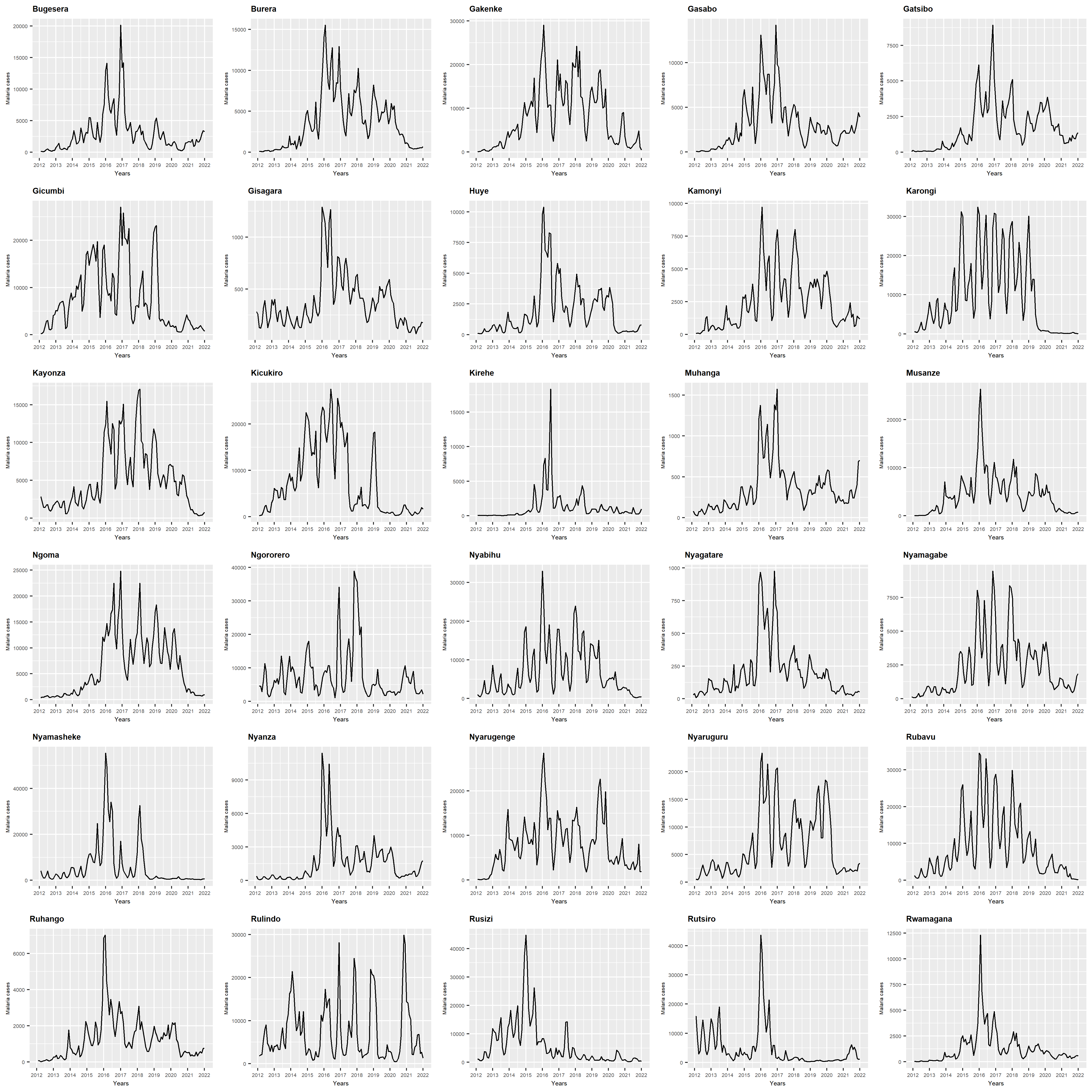

Supplement: Supplementary file 6 — Supplementary Material 6. [file 12936_2024_5097_MOESM6_ESM.png]

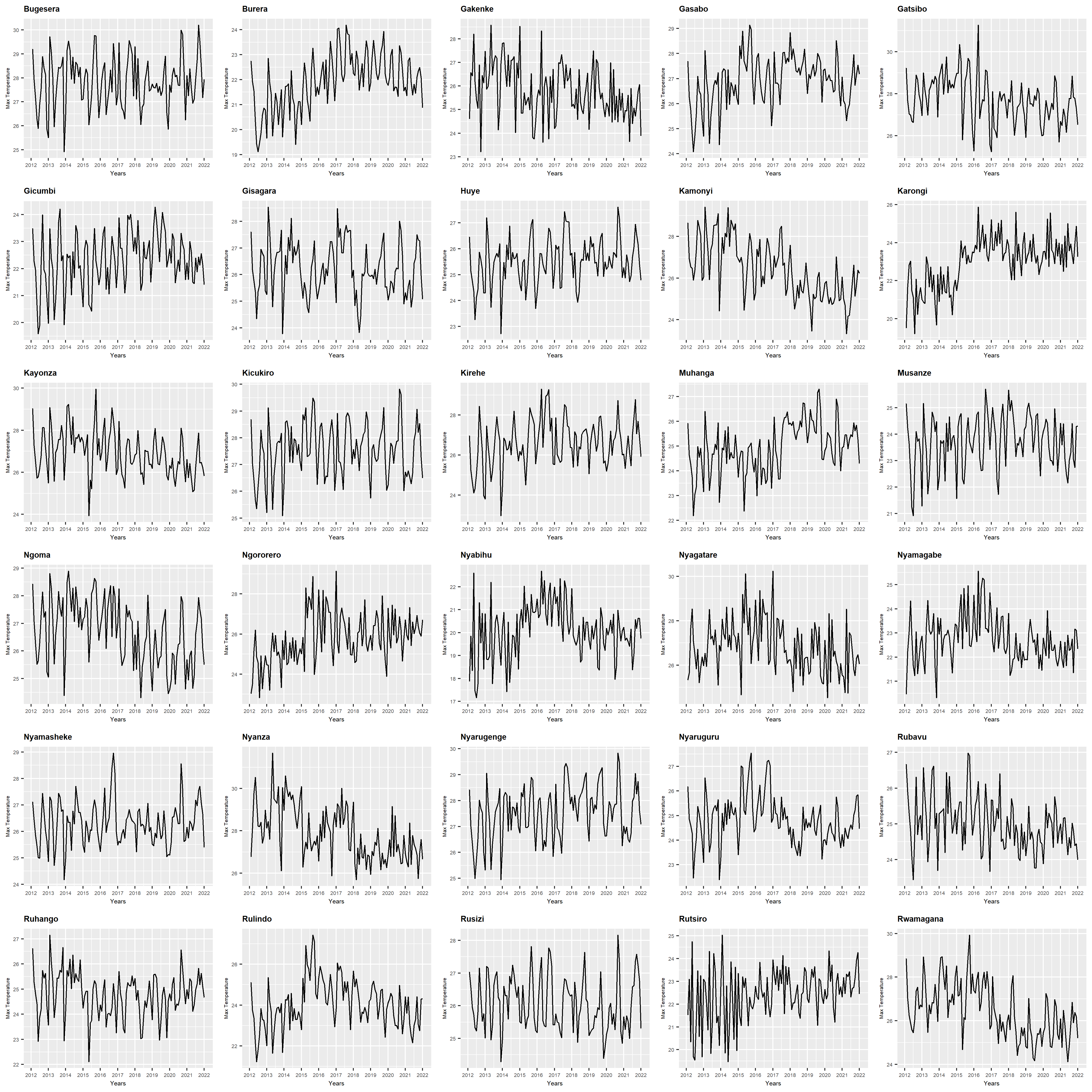

Supplement: Supplementary file 7 — Supplementary Material 7. [file 12936_2024_5097_MOESM7_ESM.png]

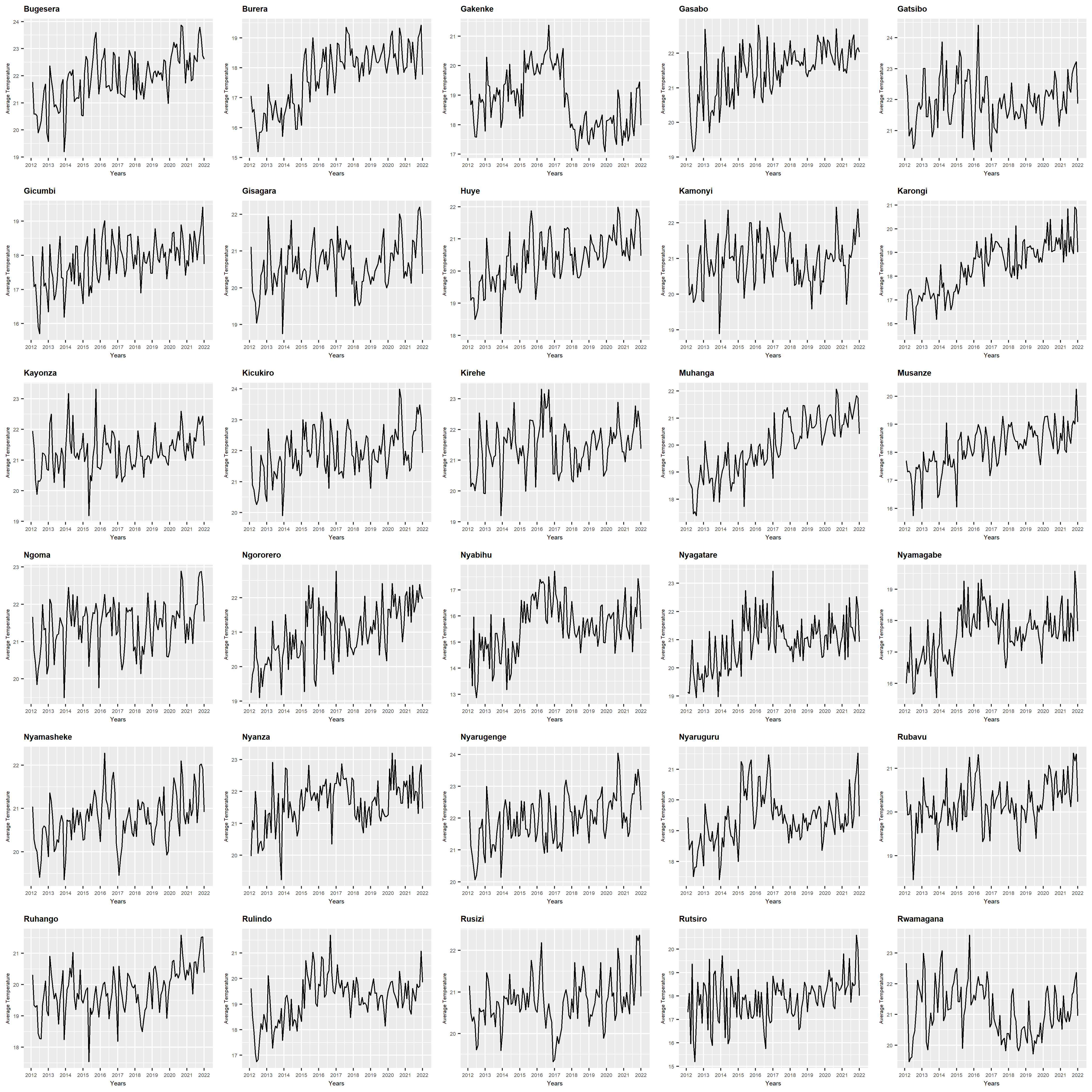

Supplement: Supplementary file 8 — Supplementary Material 8. [file 12936_2024_5097_MOESM8_ESM.png]

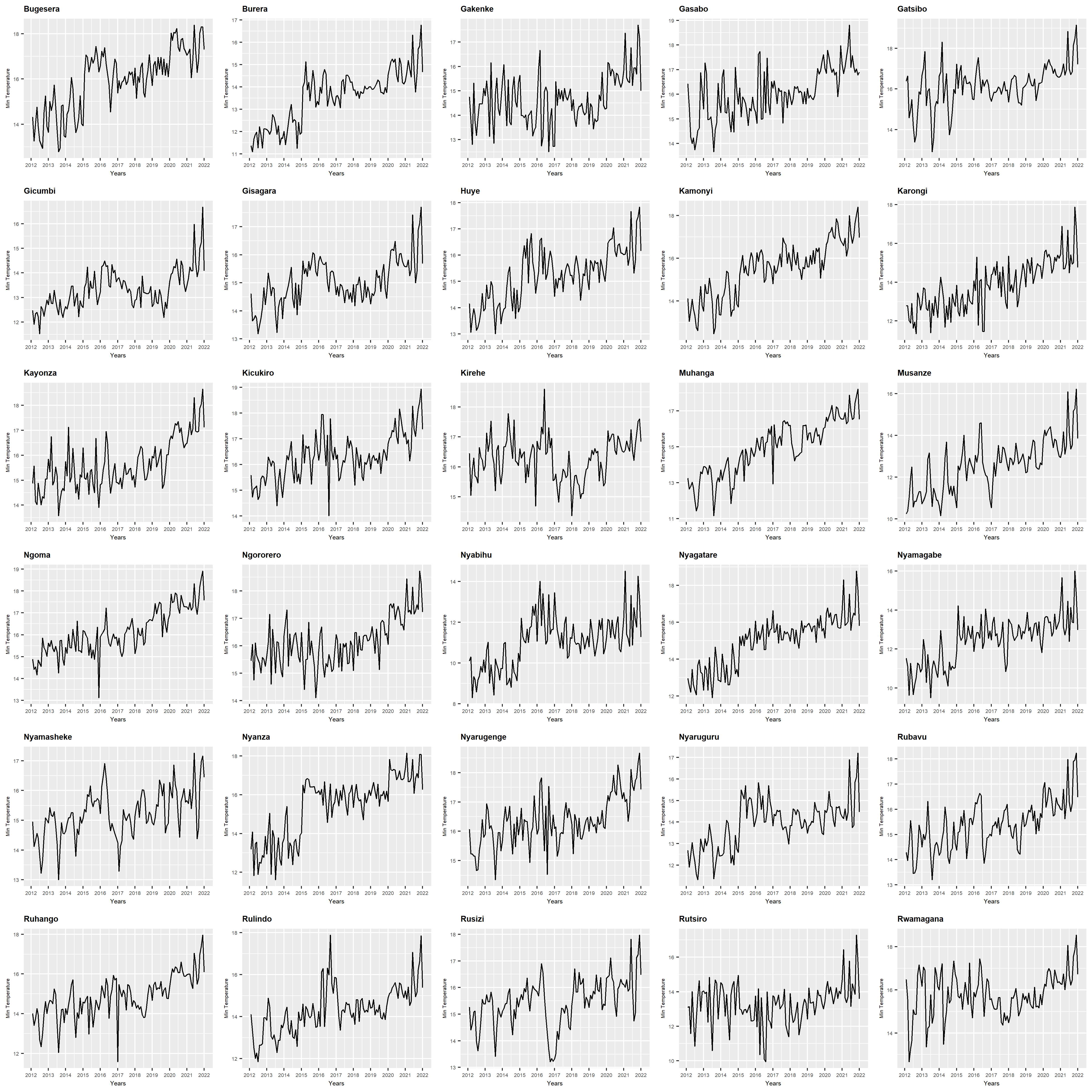

Supplement: Supplementary file 9 — Supplementary Material 9. [file 12936_2024_5097_MOESM9_ESM.png]

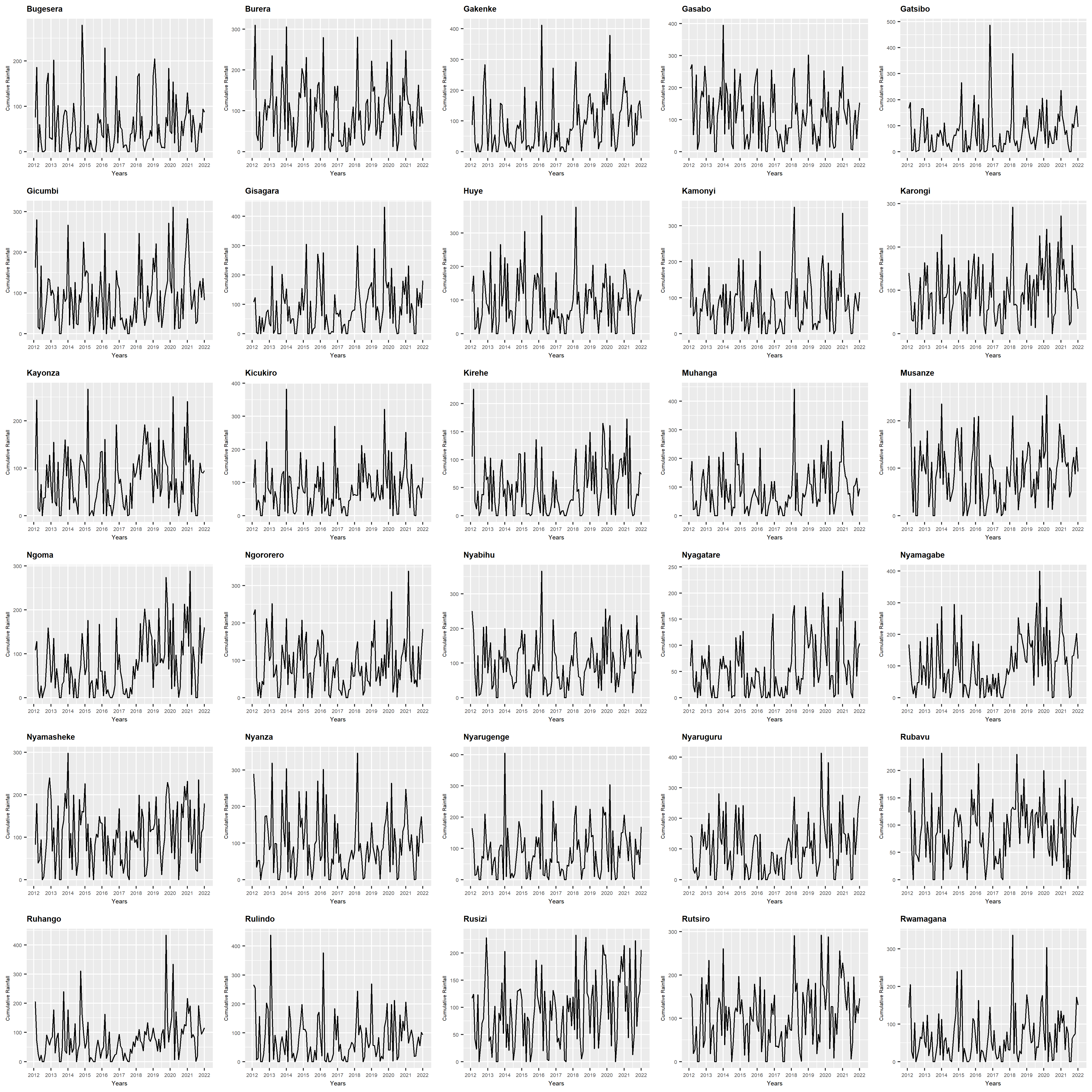

Supplement: Supplementary file 10 — Supplementary Material 10. [file 12936_2024_5097_MOESM10_ESM.png]

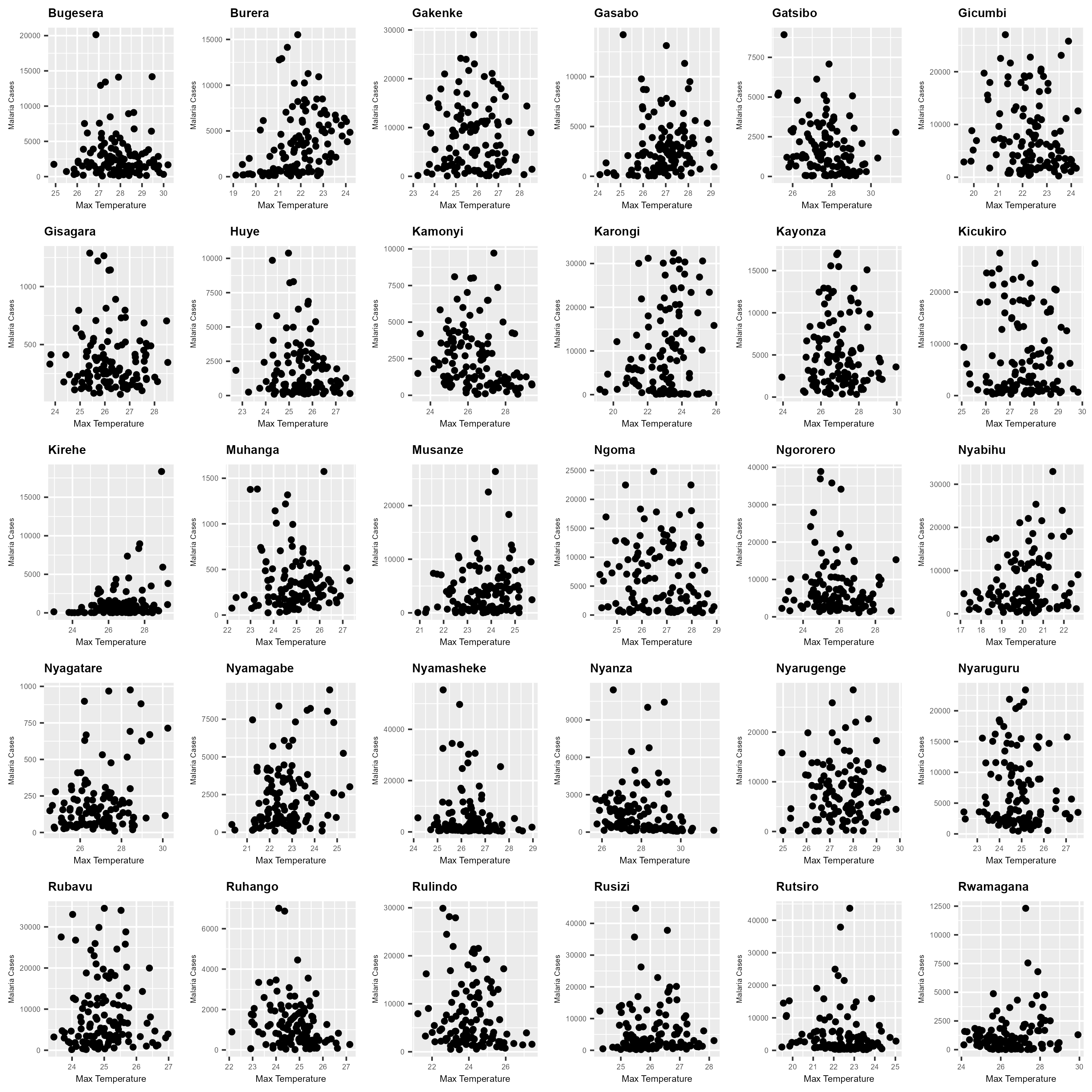

Supplement: Supplementary file 11 — Supplementary Material 11. [file 12936_2024_5097_MOESM11_ESM.png]

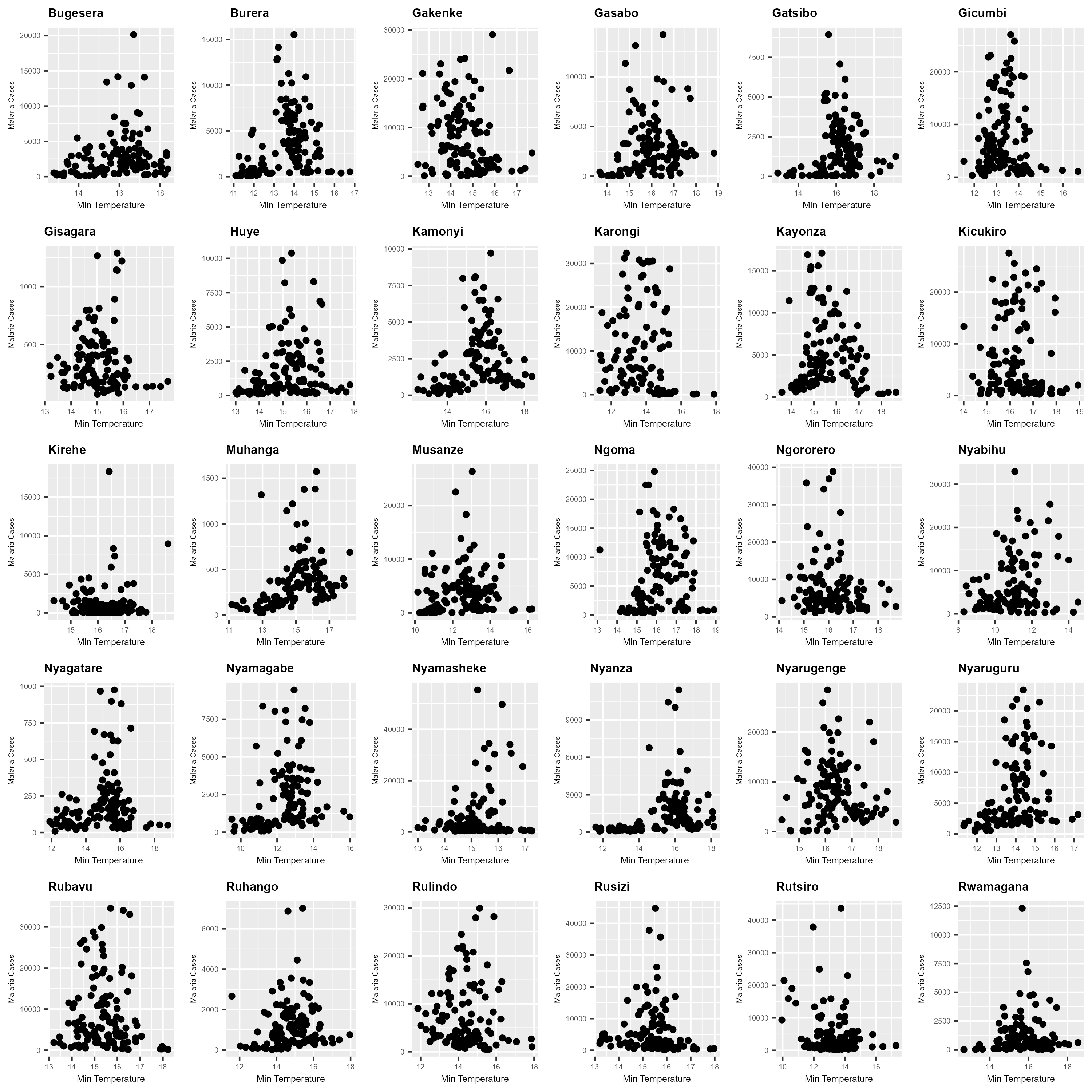

Supplement: Supplementary file 12 — Supplementary Material 12. [file 12936_2024_5097_MOESM12_ESM.png]

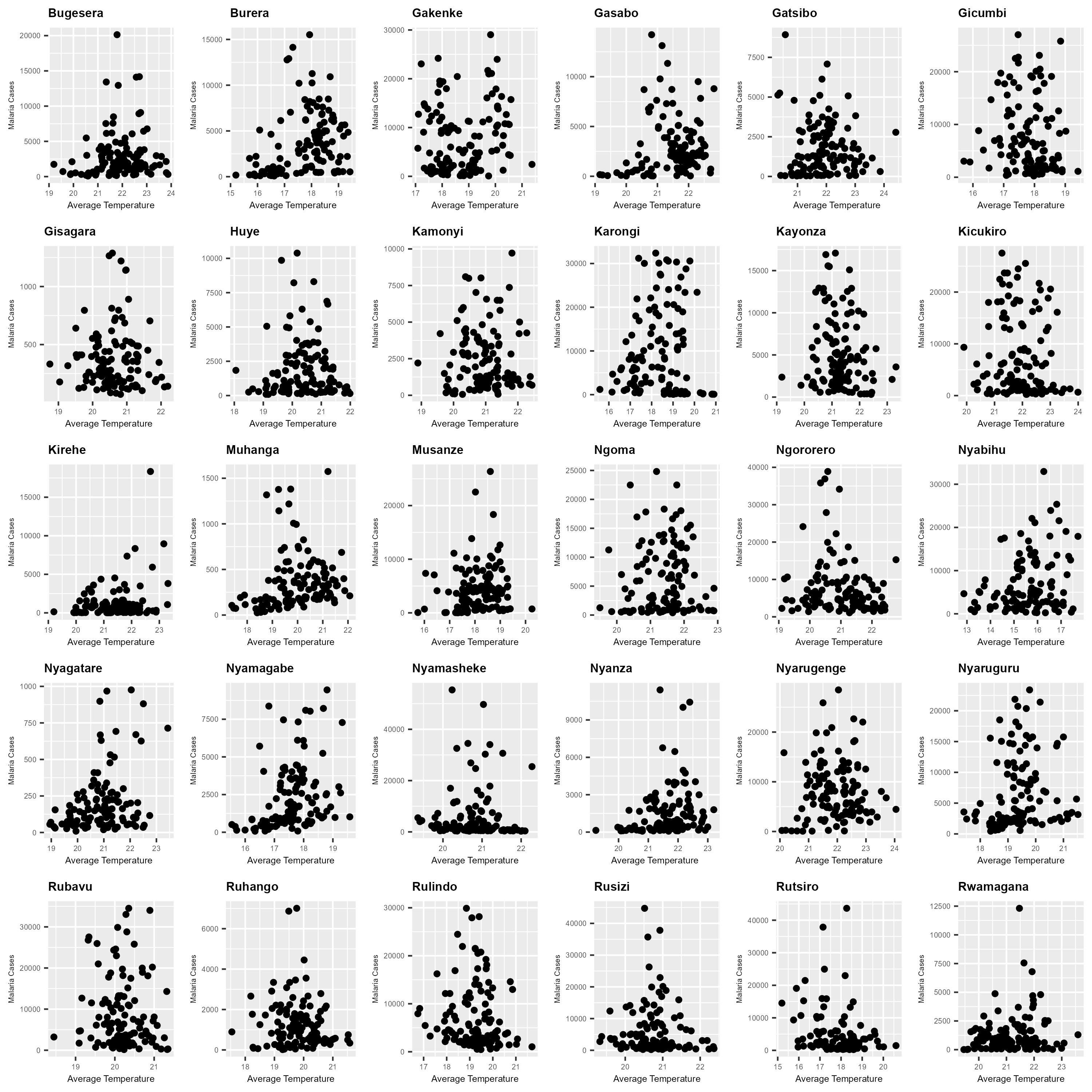

Supplement: Supplementary file 13 — Supplementary Material 13. [file 12936_2024_5097_MOESM13_ESM.png]

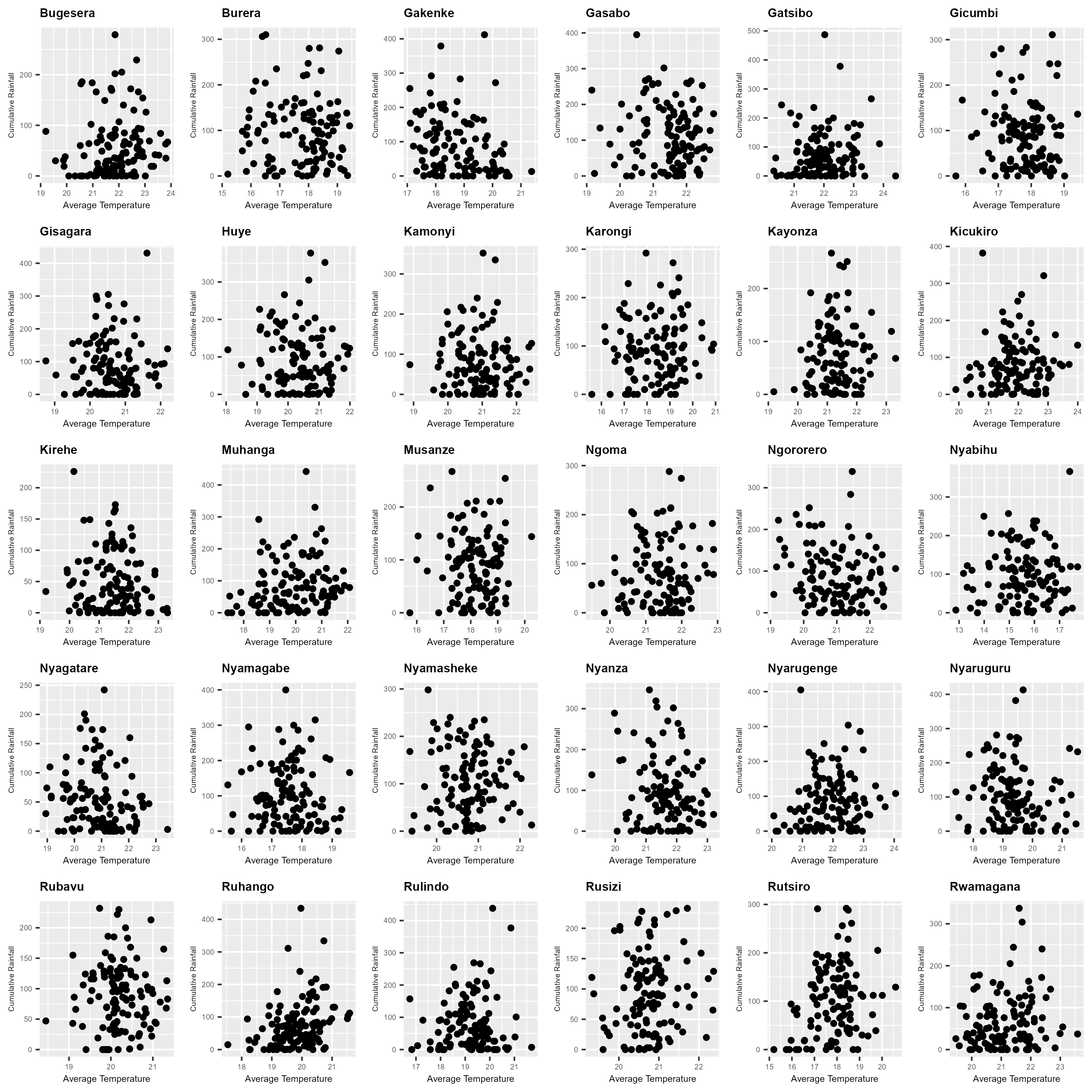

Supplement: Supplementary file 14 — Supplementary Material 14. [file 12936_2024_5097_MOESM14_ESM.png]

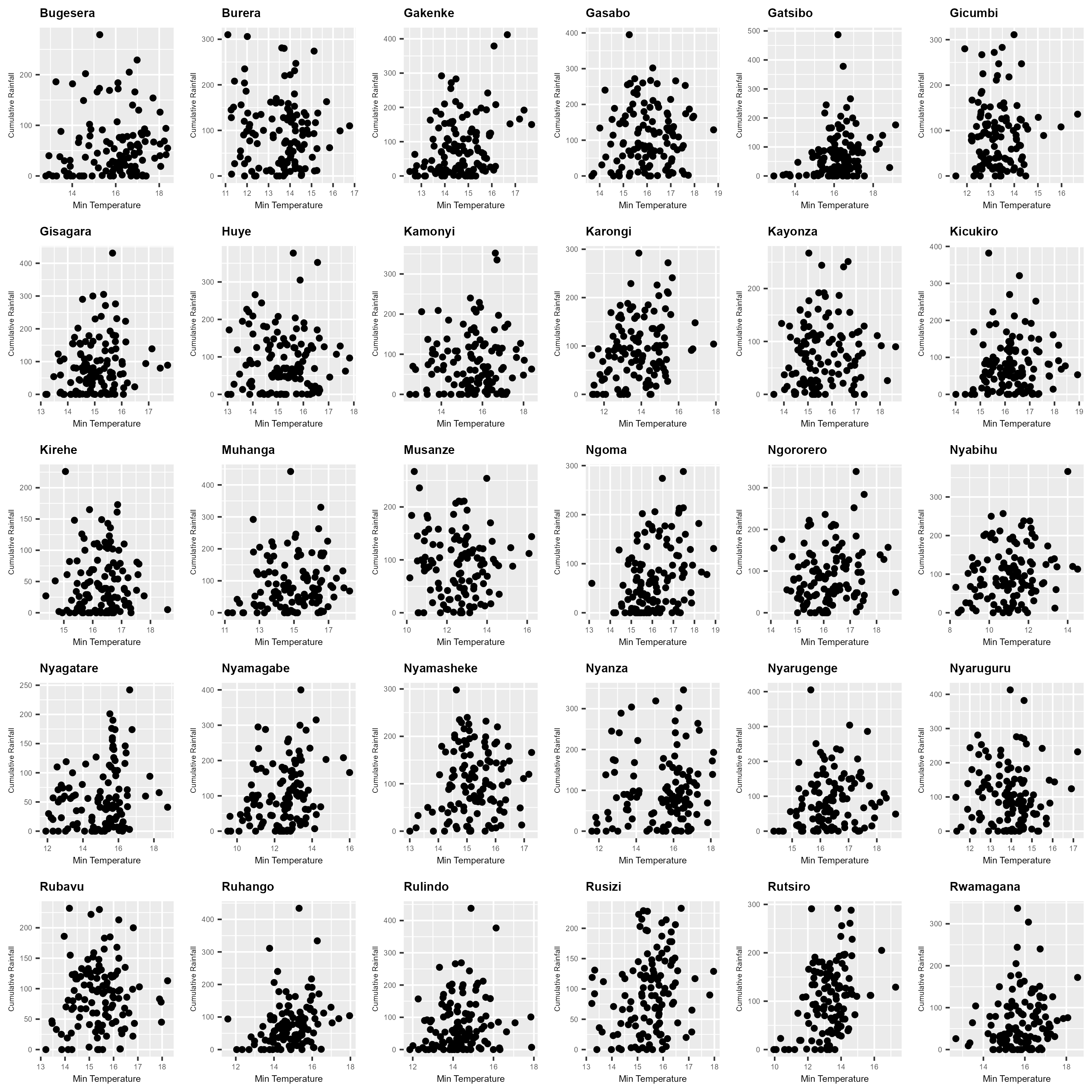

Supplement: Supplementary file 15 — Supplementary Material 15. [file 12936_2024_5097_MOESM15_ESM.png]

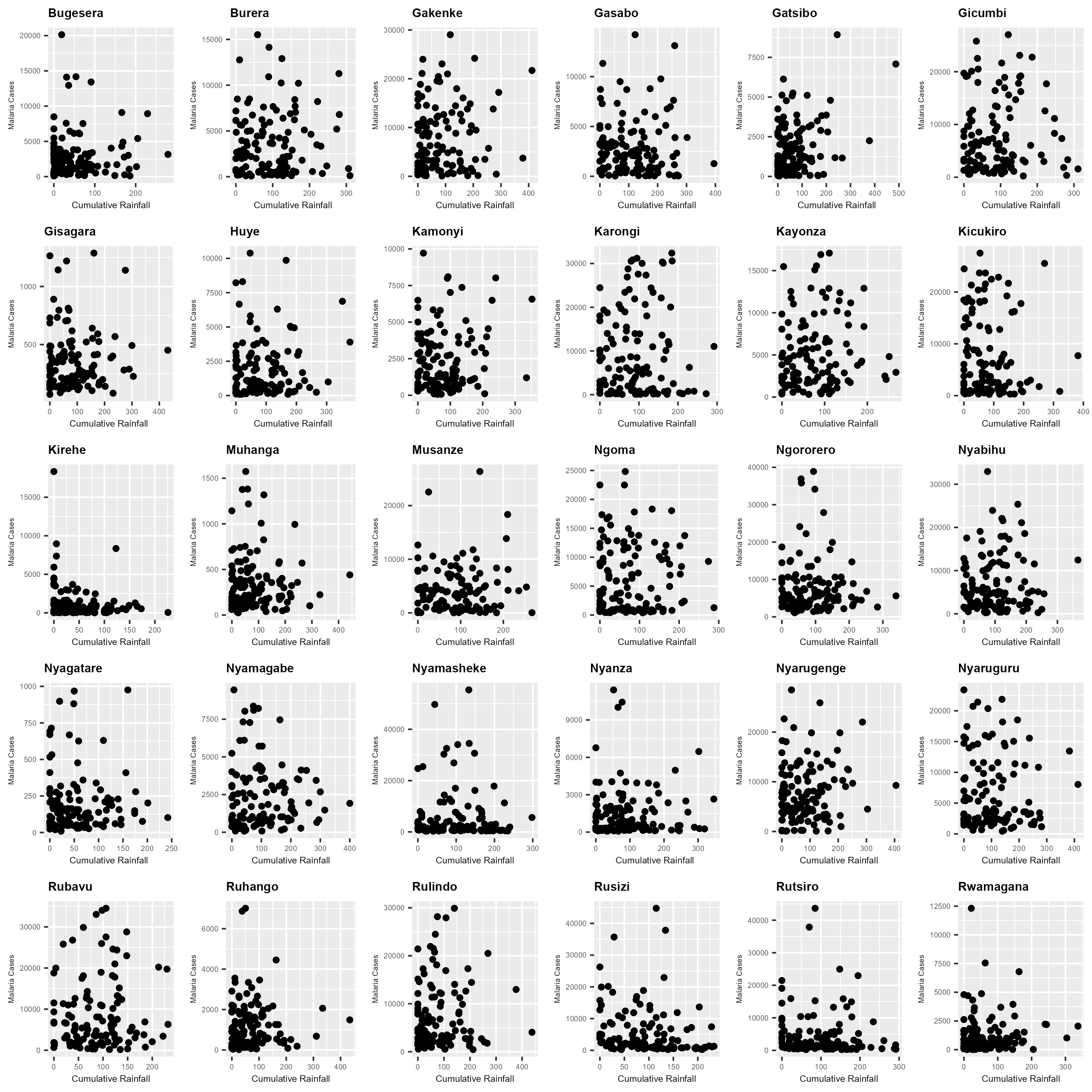

Supplement: Supplementary file 16 — Supplementary Material 16. [file 12936_2024_5097_MOESM16_ESM.png]

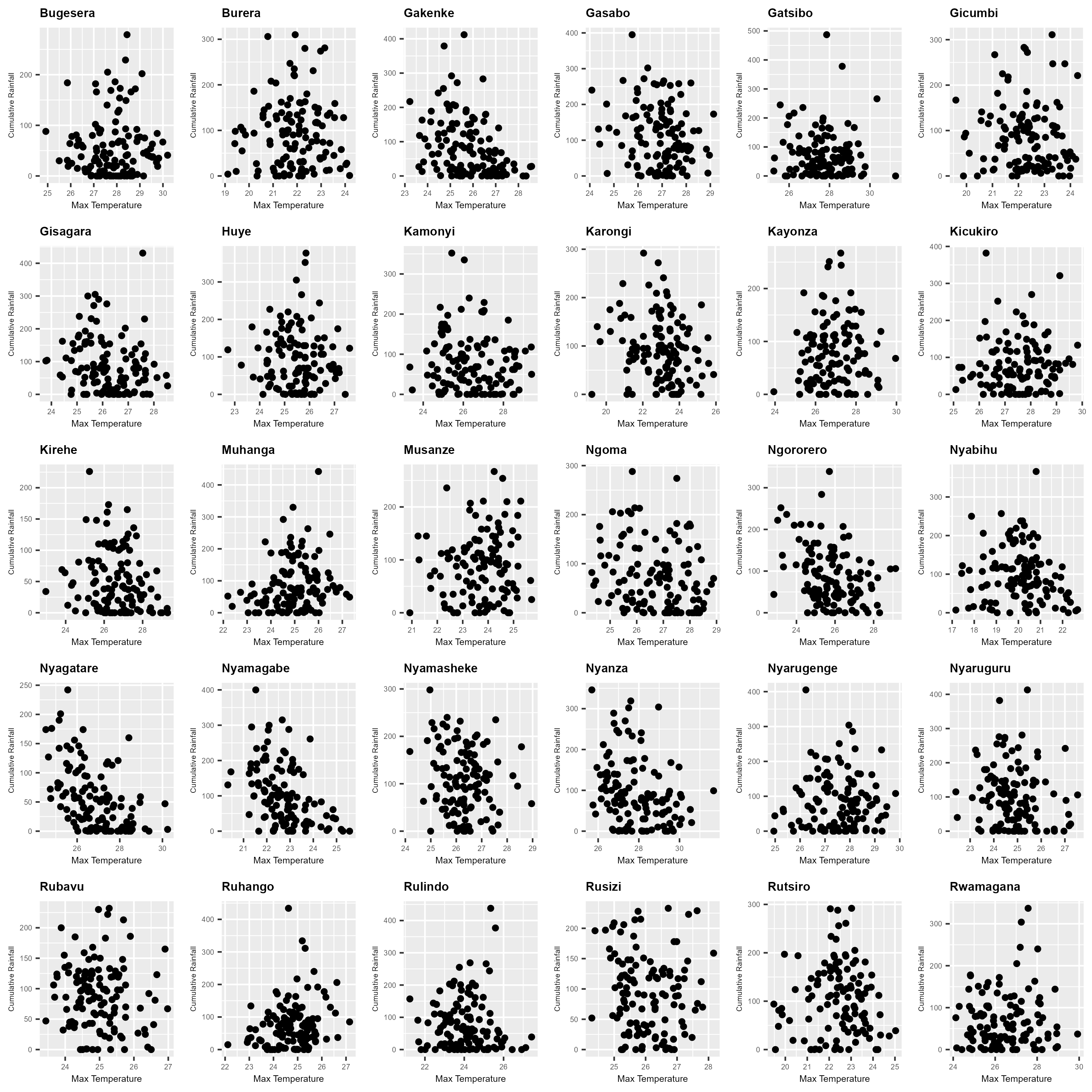

Supplement: Supplementary file 17 — Supplementary Material 17. [file 12936_2024_5097_MOESM17_ESM.png]

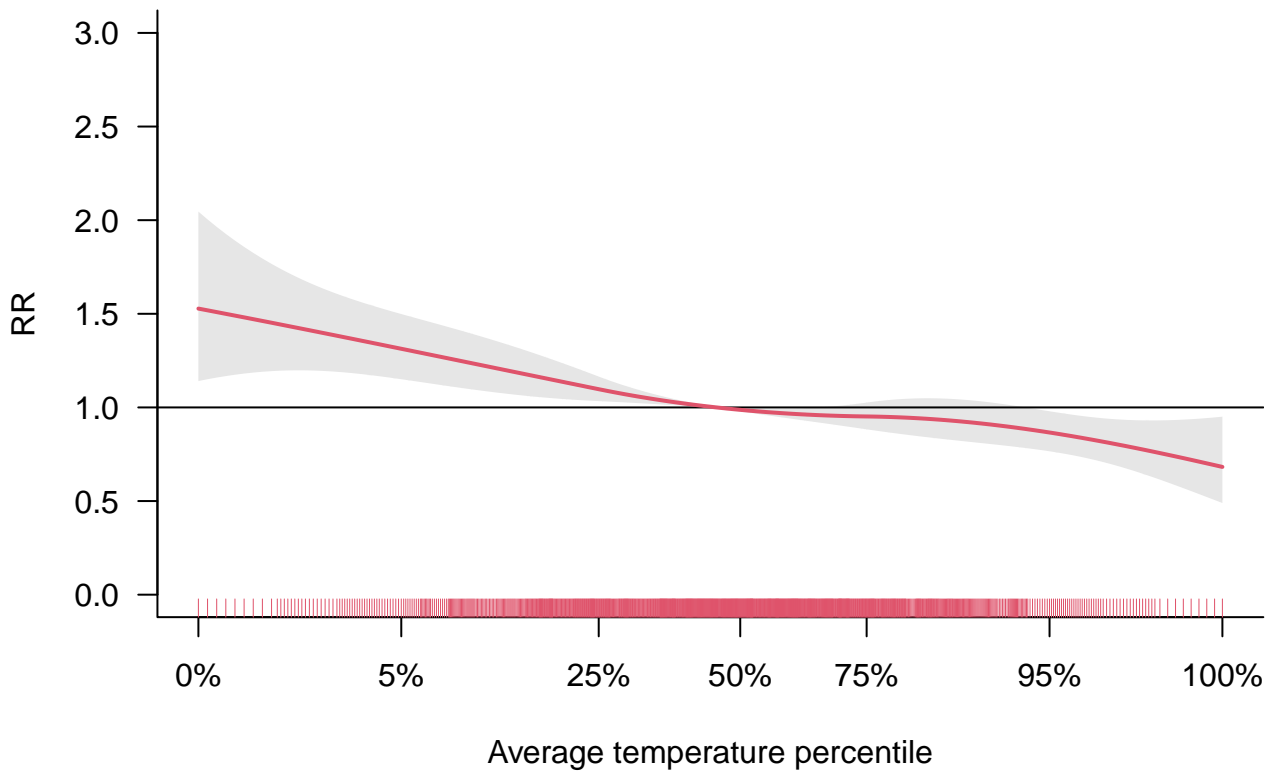

Supplement: Supplementary file 25 — Supplementary Material 25. [file 12936_2024_5097_MOESM25_ESM.pdf]

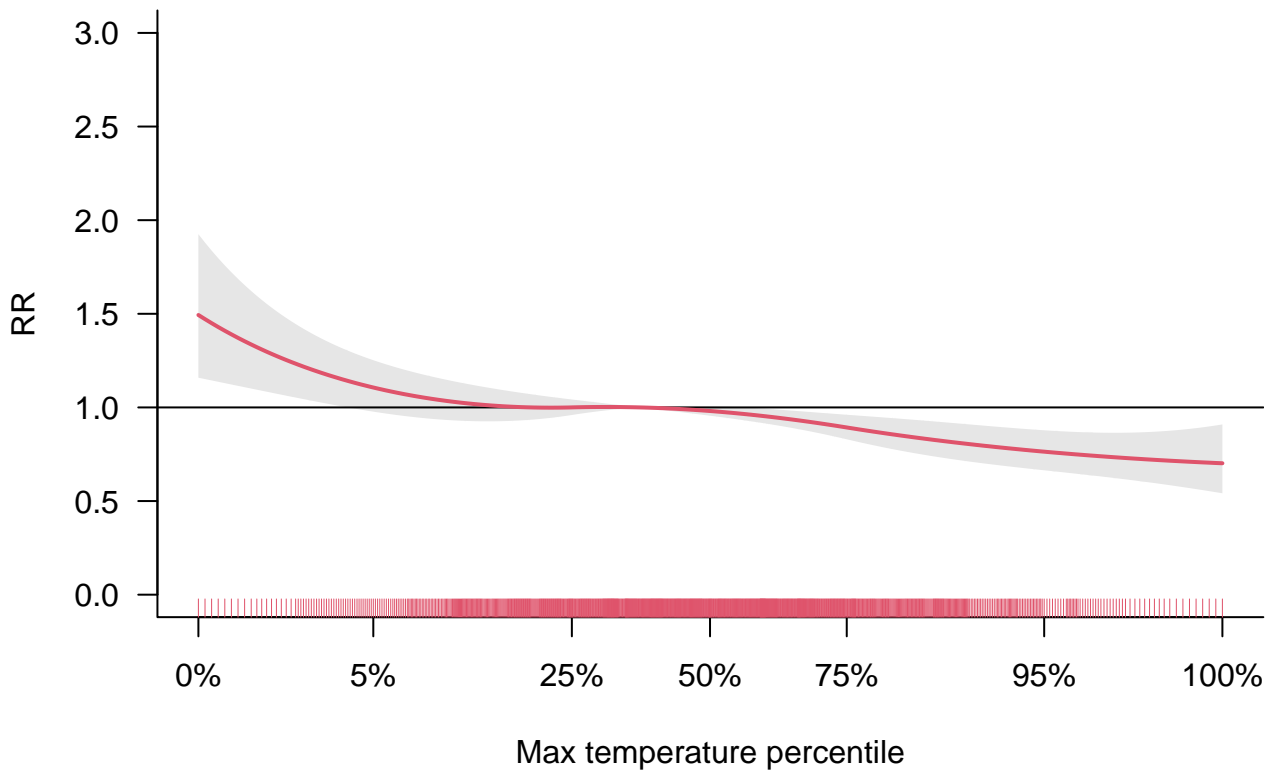

Supplement: Supplementary file 26 — Supplementary Material 26. [file 12936_2024_5097_MOESM26_ESM.pdf]

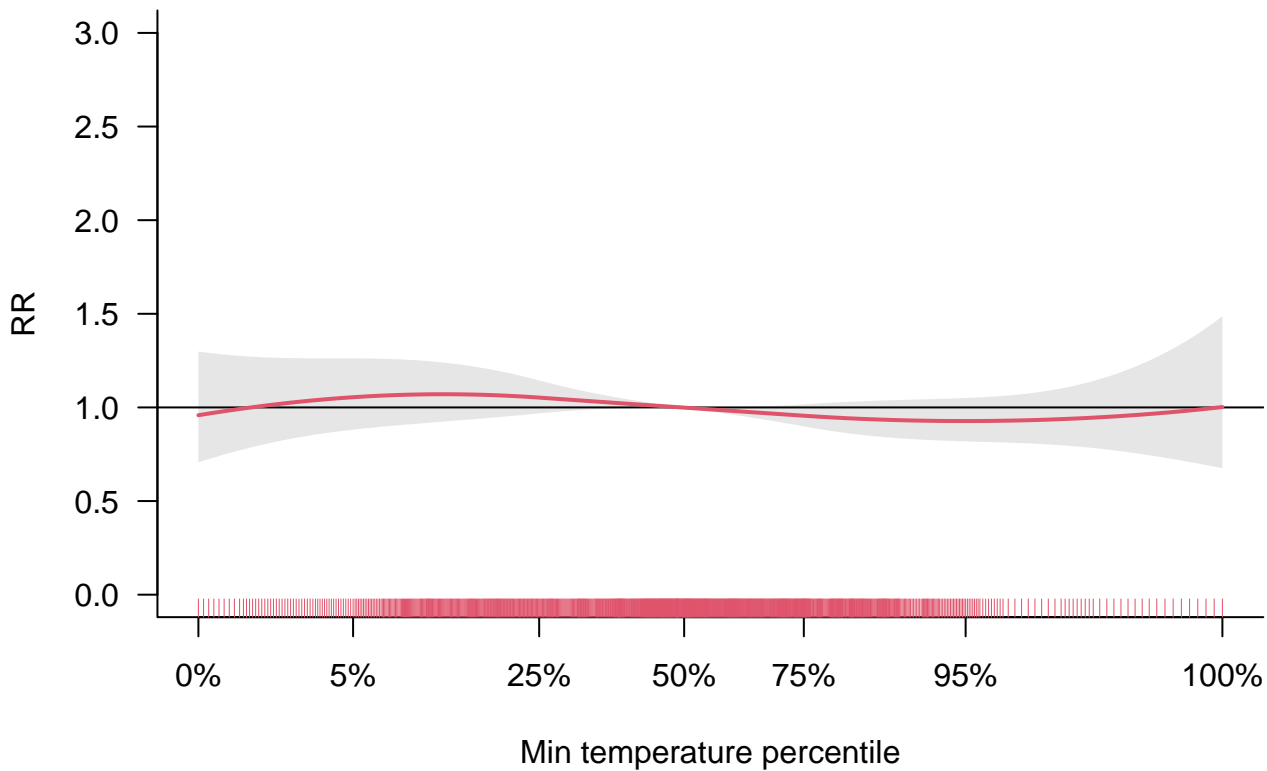

Supplement: Supplementary file 27 — Supplementary Material 27. [file 12936_2024_5097_MOESM27_ESM.pdf]

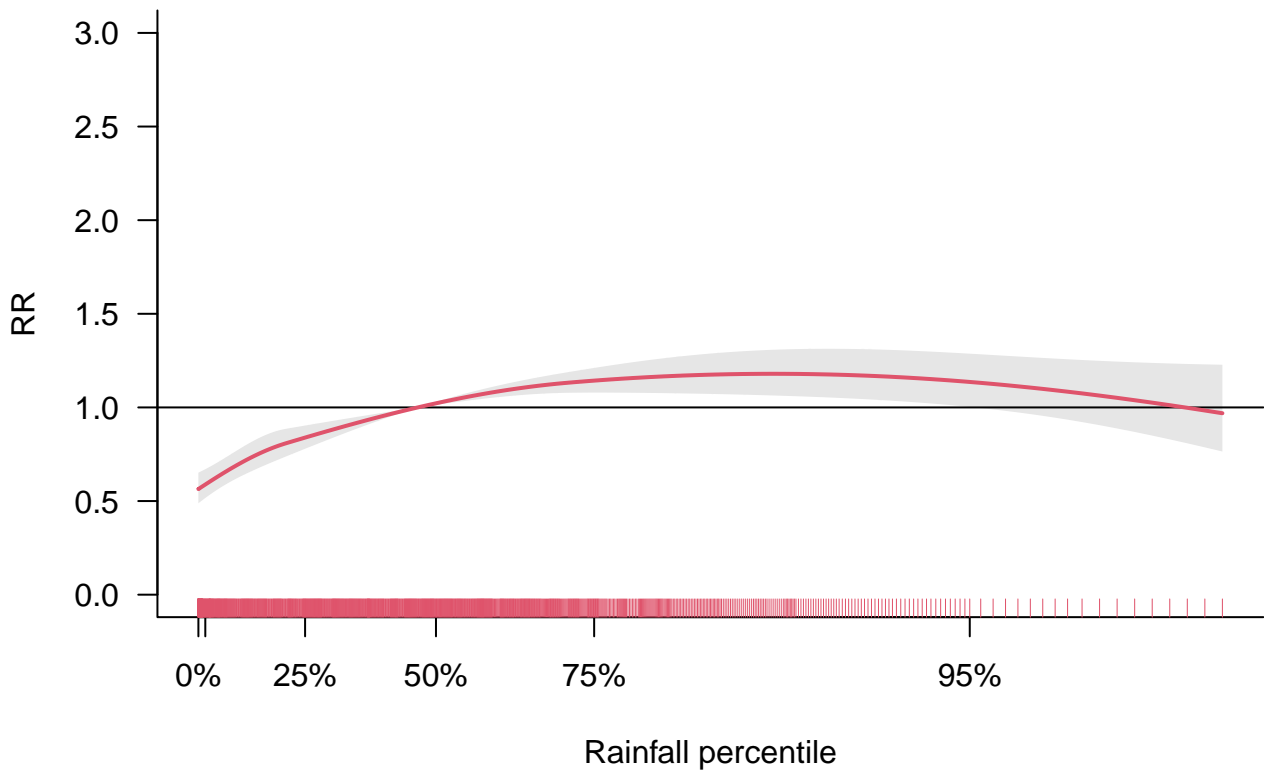

Supplement: Supplementary file 30 — Supplementary Material 30. [file 12936_2024_5097_MOESM30_ESM.pdf]

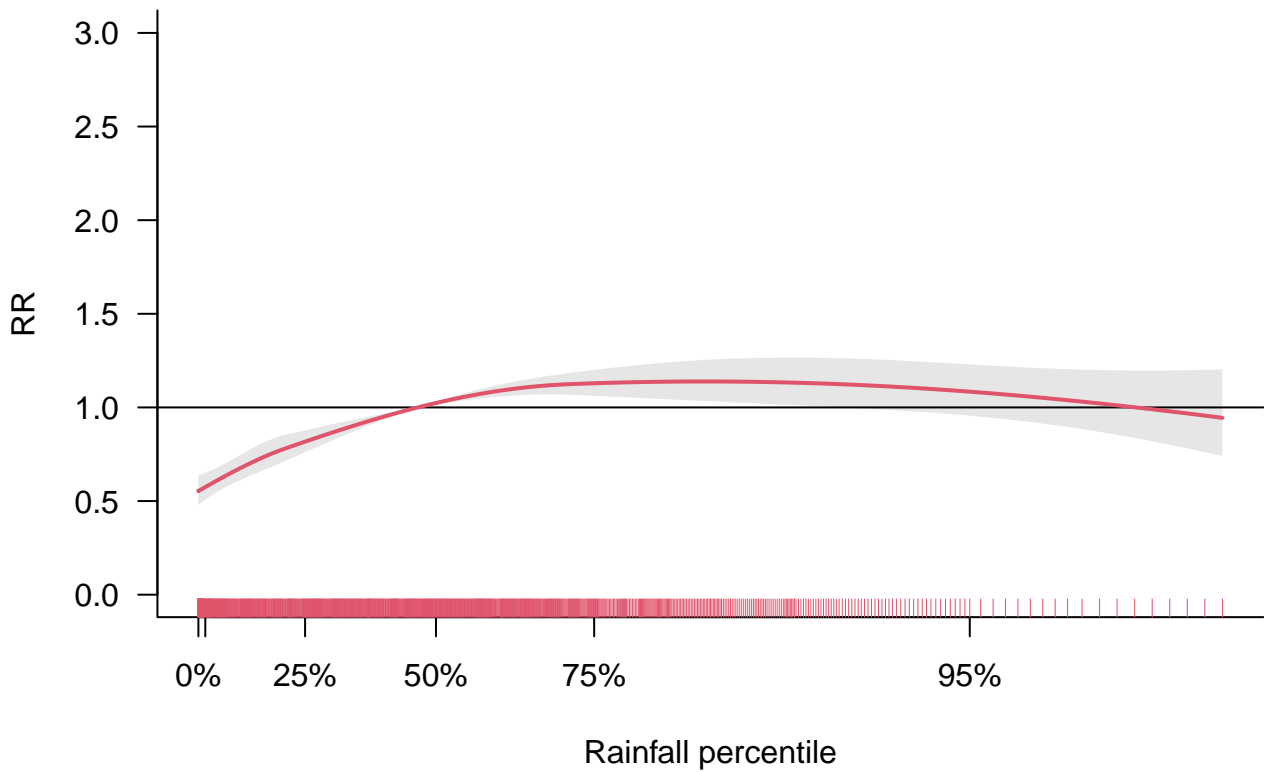

Supplement: Supplementary file 31 — Supplementary Material 31. [file 12936_2024_5097_MOESM31_ESM.pdf]

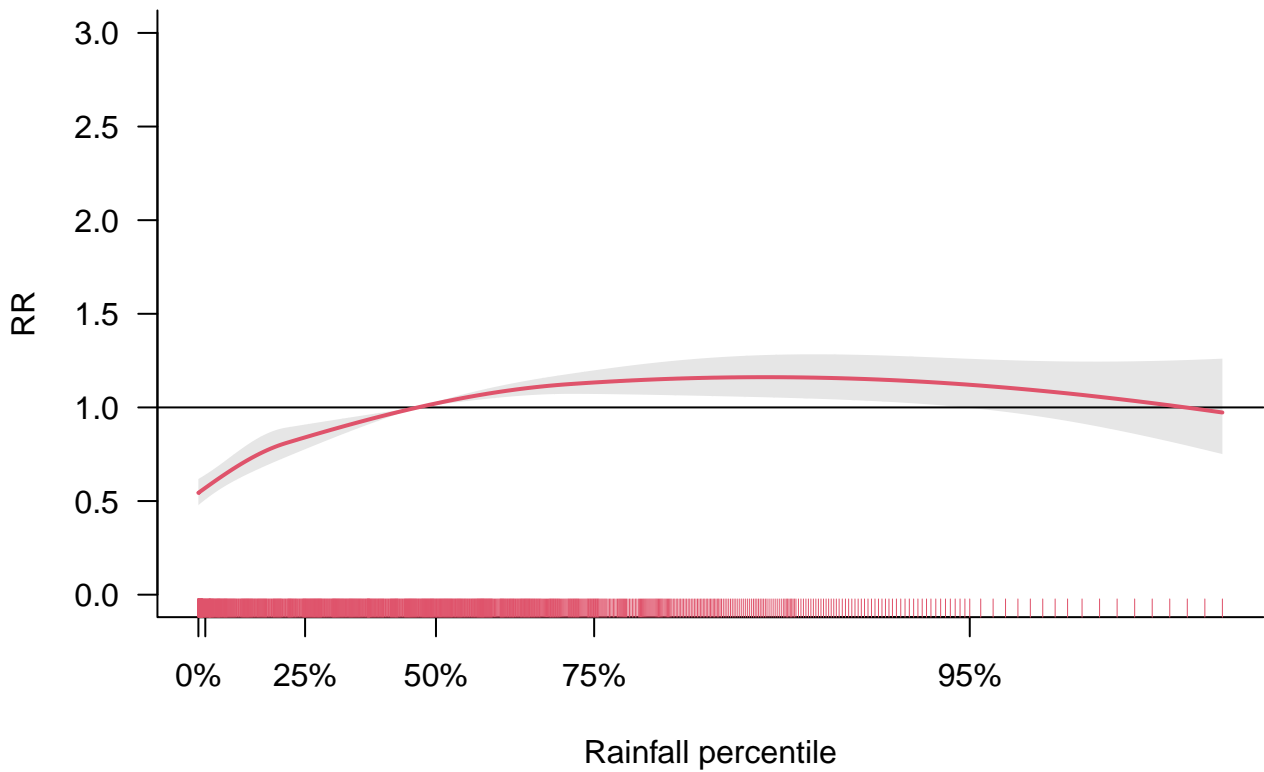

Supplement: Supplementary file 33 — Supplementary Material 33. [file 12936_2024_5097_MOESM33_ESM.pdf]
