# Supplementary material for: Potential impact of climatic factors on malaria in Rwanda between 2012 and 2021: a time-series analysis
Source: Malar J. 2024 Sep 10;23:274. doi: 10.1186/s12936-024-05097-5 (PMC11389490; doi:10.1186/s12936-024-05097-5)
Supplement: Supplementary file 24 — Supplementary Material 24. [file 12936_2024_5097_MOESM24_ESM.pdf]

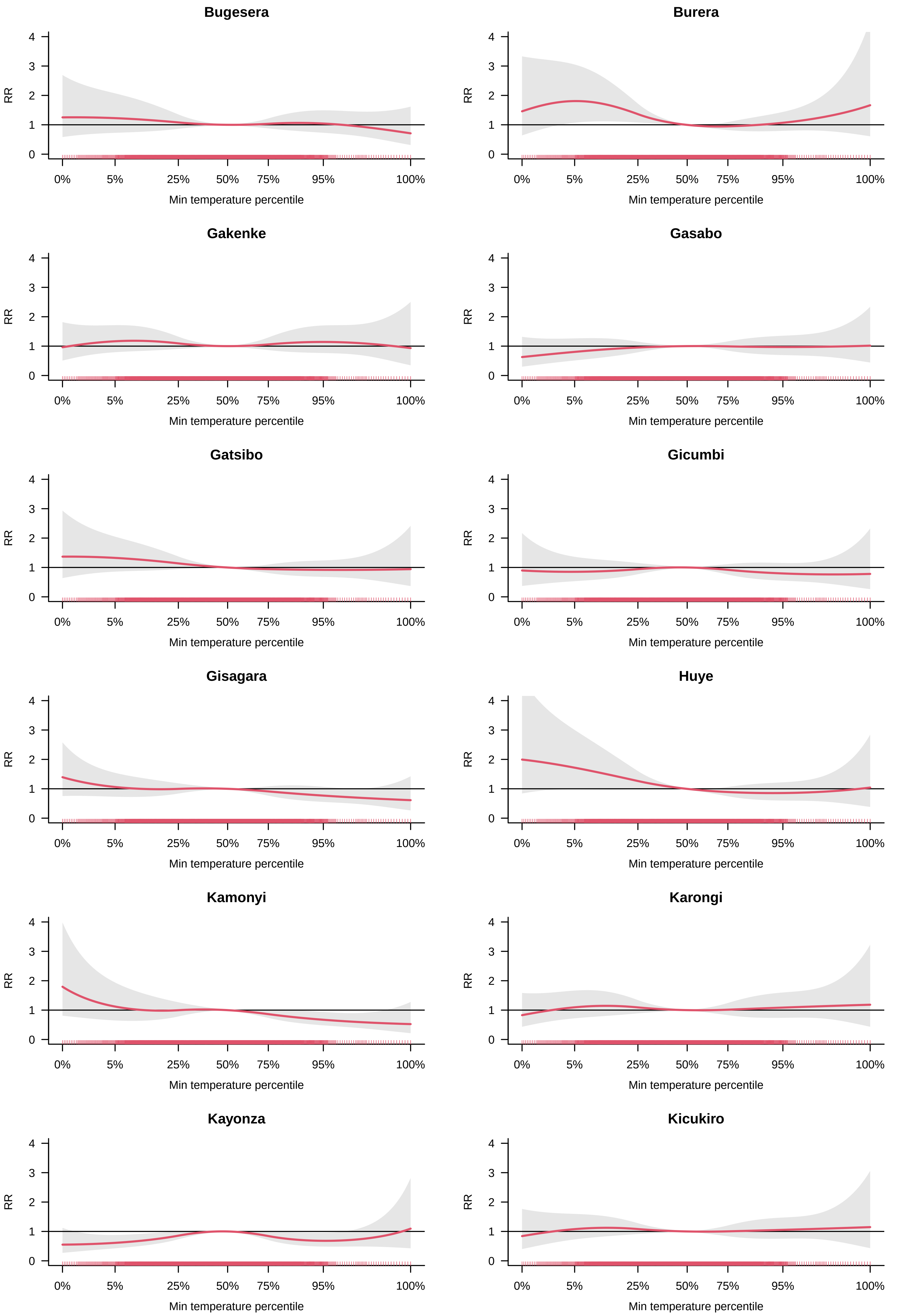
Bugesera
Burera
Gakenke
Gasabo
Gatsibo
Gicumbi
Gisagara
Huye
Kamonyi
Karongi
Kayonza
Kicukiro
RR
0 1 2 3 4
0% 5% 25% 50% 75% 95% 100%
Min temperature percentile

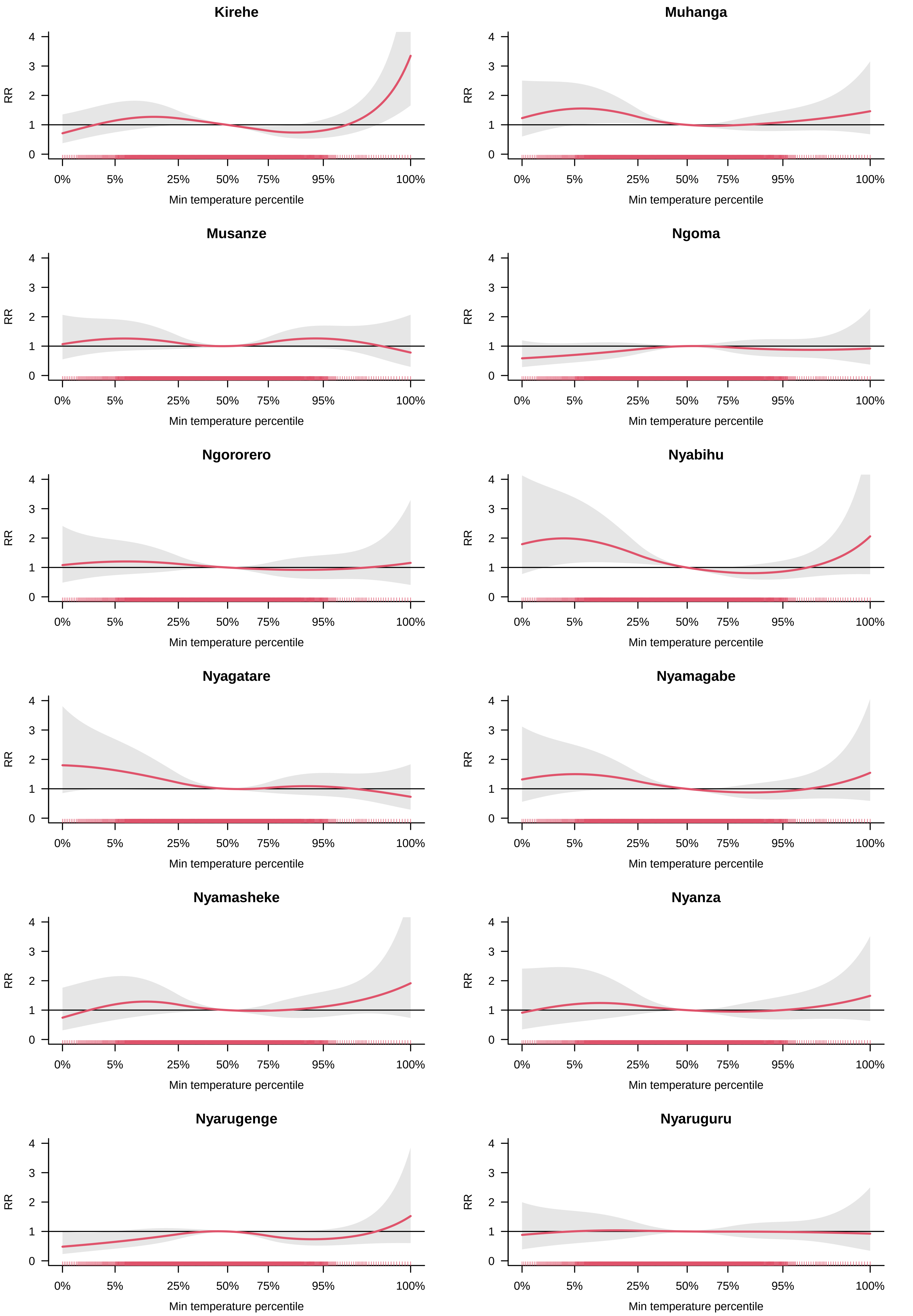
Kirehe
Muhanga
Musanze
Ngoma
Ngororero
Nyabihu
Nyagatare
Nyamagabe
Nyamasheke
Nyanza
Nyarugenge
Nyaruguru
RR
0
1
2
3
4
0%
5%
25%
50%
75%
95%
100%
Min temperature percentile

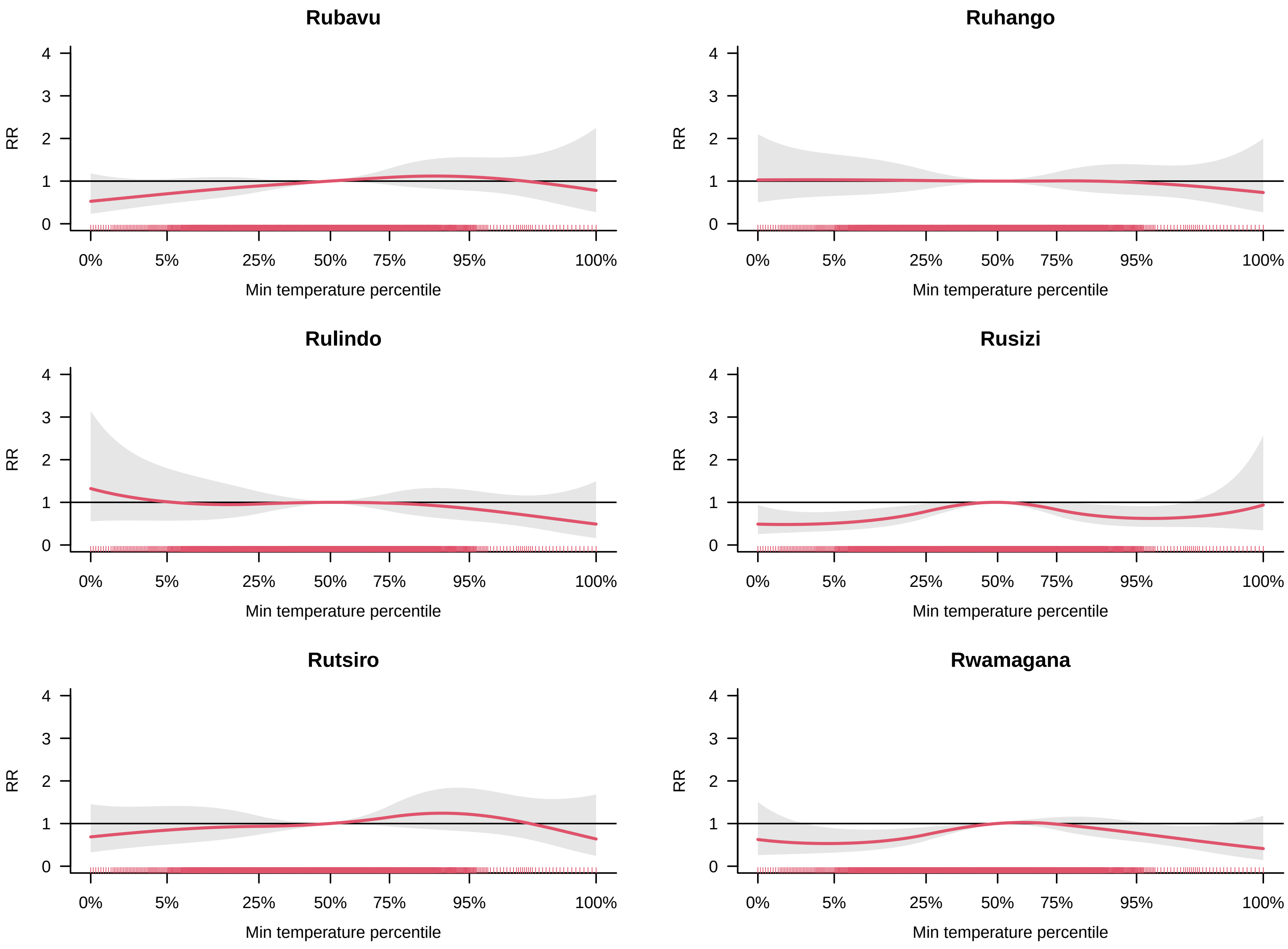

Rubavu
Ruhango
Rulindo
Rusizi
Rutsiro
Rwamagana
RR
0
1
2
3
4
0%
5%
25%
50%
75%
95%
100%
Min temperature percentile
